# Supplementary material for: Effect of semen dilution rate and dimethyl acetamide levels on post-thaw motility and fertility parameters of rooster sperm
Source: PLoS One. 2025 Oct 31;20(10):e0335748. doi: 10.1371/journal.pone.0335748 (PMC12578245; doi:10.1371/journal.pone.0335748)
Supplement: S1 File — (PDF) [file pone.0335748.s001.pdf]

	Pool	field	Interaction	Group	DMA	TM
1	1	1	LC3	LC	3	43.36
2	1	2	LC3	LC	3	44.21
3	1	3	LC3	LC	3	39.01
4	1	4	LC3	LC	3	45.66
5	1	5	LC3	LC	3	43.06
6	2	1	LC3	LC	3	44.66
7	2	2	LC3	LC	3	43.36
8	2	3	LC3	LC	3	44.21
9	2	4	LC3	LC	3	39.01
10	2	5	LC3	LC	3	42.81
11	3	1	LC3	LC	3	45.66
12	3	2	LC3	LC	3	44.66
13	3	3	LC3	LC	3	36.68
14	3	4	LC3	LC	3	40.11
15	3	5	LC3	LC	3	41.78
16	4	1	LC3	LC	3	36.36
17	4	2	LC3	LC	3	36.03
18	4	3	LC3	LC	3	33.33
19	4	4	LC3	LC	3	43.36
20	4	5	LC3	LC	3	44.21
21	4	6	LC3	LC	3	38.66
22	5	1	LC3	LC	3	39.01
23	5	2	LC3	LC	3	45.66
24	5	3	LC3	LC	3	44.66
25	5	4	LC3	LC	3	36.68
26	5	5	LC3	LC	3	40.11
27	5	6	LC3	LC	3	41.22
28	6	1	LC3	LC	3	36.36
29	6	2	LC3	LC	3	36.03
30	6	3	LC3	LC	3	33.33
31	6	4	LC3	LC	3	43.36
32	6	5	LC3	LC	3	44.21
33	6	6	LC3	LC	3	38.66
34	7	1	LC3	LC	3	39.01
35	7	2	LC3	LC	3	45.66
36	7	3	LC3	LC	3	44.66

CASA-DATA2.sav

	Prog	DAP	DCL	DSL	VAP	VCL
1	26.64	19.96	36.32	12.88	50.35	91.05
2	28.42	18.65	31.14	13.11	47.48	78.44
3	21.21	23.05	43.11	13.65	57.81	108.40
4	25.57	19.52	37.57	12.49	47.26	91.00
5	25.46	20.30	37.04	13.03	50.73	92.22
6	30.42	19.51	36.00	12.45	50.21	91.61
7	26.64	19.96	36.32	12.88	50.35	91.05
8	28.42	18.65	31.14	13.11	47.48	78.44
9	21.21	23.05	43.11	13.65	57.81	108.40
10	26.67	20.29	36.64	13.02	51.46	92.38
11	25.57	19.52	37.57	12.49	47.26	91.00
12	30.42	19.51	36.00	12.45	50.21	91.61
13	19.65	21.14	36.95	14.06	54.46	94.87
14	21.16	20.33	35.65	13.41	52.18	91.19
15	24.20	20.13	36.54	13.10	51.03	92.17
16	17.81	20.52	35.70	14.02	52.74	91.92
17	17.00	22.07	38.89	15.12	56.23	97.14
18	21.76	21.96	38.20	14.18	57.31	100.02
19	26.64	19.96	36.32	12.88	50.35	91.05
20	28.42	18.65	31.14	13.11	47.48	78.44
21	22.33	20.63	36.05	13.86	52.82	91.71
22	21.21	23.05	43.11	13.65	57.81	108.40
23	25.57	19.52	37.57	12.49	47.26	91.00
24	30.42	19.51	36.00	12.45	50.21	91.61
25	19.65	21.14	36.95	14.06	54.46	94.87
26	21.16	20.33	35.65	13.41	52.18	91.19
27	23.60	20.71	37.86	13.21	52.38	95.41
28	17.81	20.52	35.70	14.02	52.74	91.92
29	17.00	22.07	38.89	15.12	56.23	97.14
30	21.76	21.96	38.20	14.18	57.31	100.02
31	26.64	19.96	36.32	12.88	50.35	91.05
32	28.42	18.65	31.14	13.11	47.48	78.44
33	22.33	20.63	36.05	13.86	52.82	91.71
34	21.21	23.05	43.11	13.65	57.81	108.40
35	25.57	19.52	37.57	12.49	47.26	91.00
36	30.42	19.51	36.00	12.45	50.21	91.61

	VSL	STR	LIN	WOB	ALH	BCF
1	32.87	.65	.36	.55	4.43	22.50
2	33.79	.71	.43	.60	4.61	22.61
3	34.62	.59	.31	.53	4.75	24.96
4	30.40	.64	.33	.51	4.43	23.38
5	32.92	.65	.36	.55	4.56	23.36
6	32.50	.64	.35	.54	4.08	20.43
7	32.87	.65	.36	.55	4.43	22.50
8	33.79	.71	.43	.60	4.61	22.61
9	34.62	.59	.31	.53	4.75	24.96
10	33.45	.65	.36	.56	4.47	22.63
11	30.40	.64	.33	.51	4.43	23.38
12	32.50	.64	.35	.54	4.08	20.43
13	36.38	.66	.38	.57	4.47	26.93
14	34.62	.66	.37	.57	4.26	26.03
15	33.48	.65	.36	.55	4.31	24.19
16	36.21	.68	.39	.57	4.53	26.75
17	39.11	.69	.40	.57	4.21	26.98
18	36.80	.64	.36	.57	4.85	28.08
19	32.87	.65	.36	.55	4.43	22.50
20	33.79	.71	.43	.60	4.61	22.61
21	35.76	.67	.39	.57	4.53	25.38
22	34.62	.59	.31	.53	4.75	24.96
23	30.40	.64	.33	.51	4.43	23.38
24	32.50	.64	.35	.54	4.08	20.43
25	36.38	.66	.38	.57	4.47	26.93
26	34.62	.66	.37	.57	4.26	26.03
27	33.70	.64	.35	.54	4.40	24.35
28	36.21	.68	.39	.57	4.53	26.75
29	39.11	.69	.40	.57	4.21	26.98
30	36.80	.64	.36	.57	4.85	28.08
31	32.87	.65	.36	.55	4.43	22.50
32	33.79	.71	.43	.60	4.61	22.61
33	35.76	.67	.39	.57	4.53	25.38
34	34.62	.59	.31	.53	4.75	24.96
35	30.40	.64	.33	.51	4.43	23.38
36	32.50	.64	.35	.54	4.08	20.43

	Pool	field	Interaction	Group	DMA	TM
37	7	4	LC3	LC	3	36.68
38	7	5	LC3	LC	3	41.50
39	8	1	LC3	LC	3	40.11
40	8	2	LC3	LC	3	36.36
41	8	3	LC3	LC	3	36.03
42	8	4	LC3	LC	3	33.33
43	8	5	LC3	LC	3	36.46
44	1	1	LC6	LC	6	52.09
45	1	2	LC6	LC	6	57.44
46	1	3	LC6	LC	6	46.62
47	1	4	LC6	LC	6	49.01
48	1	5	LC6	LC	6	51.29
49	2	1	LC6	LC	6	43.20
50	2	2	LC6	LC	6	49.76
51	2	3	LC6	LC	6	47.95
52	2	4	LC6	LC	6	52.09
53	2	5	LC6	LC	6	48.25
54	3	1	LC6	LC	6	57.44
55	3	2	LC6	LC	6	46.62
56	3	3	LC6	LC	6	49.01
57	3	4	LC6	LC	6	43.20
58	3	5	LC6	LC	6	49.76
59	3	6	LC6	LC	6	49.21
60	4	1	LC6	LC	6	47.95
61	4	2	LC6	LC	6	59.31
62	4	3	LC6	LC	6	40.70
63	4	4	LC6	LC	6	52.09
64	4	5	LC6	LC	6	50.01
65	5	1	LC6	LC	6	57.44
66	5	2	LC6	LC	6	46.62
67	5	3	LC6	LC	6	49.01
68	5	4	LC6	LC	6	43.20
69	5	5	LC6	LC	6	49.76
70	5	6	LC6	LC	6	49.21
71	6	1	LC6	LC	6	47.95
72	6	2	LC6	LC	6	59.31

	Prog	DAP	DCL	DSL	VAP	VCL
37	19.65	21.14	36.95	14.06	54.46	94.87
38	24.21	20.81	38.41	13.16	52.44	96.47
39	21.16	20.33	35.65	13.41	52.18	91.19
40	17.81	20.52	35.70	14.02	52.74	91.92
41	17.00	22.07	38.89	15.12	56.23	97.14
42	21.76	21.96	38.20	14.18	57.31	100.02
43	19.43	21.22	37.11	14.18	54.62	95.07
44	35.19	20.72	34.27	13.81	51.24	83.67
45	39.14	21.06	35.10	14.01	51.48	85.06
46	31.15	20.29	33.20	13.55	50.94	81.87
47	32.94	23.83	41.94	15.97	60.33	105.93
48	34.61	21.48	36.13	14.34	53.50	89.13
49	28.80	23.05	40.56	16.61	55.26	97.36
50	34.88	23.93	41.43	16.11	58.92	101.49
51	38.93	26.10	41.99	18.27	65.85	106.07
52	35.19	20.72	34.27	13.81	51.24	83.67
53	34.45	23.45	39.56	16.20	57.82	97.15
54	39.14	21.06	35.10	14.01	51.48	85.06
55	31.15	20.29	33.20	13.55	50.94	81.87
56	32.94	23.83	41.94	15.97	60.33	105.93
57	28.80	23.05	40.56	16.61	55.26	97.36
58	34.88	23.93	41.43	16.11	58.92	101.49
59	33.38	22.43	38.45	15.25	55.39	94.34
60	38.93	26.10	41.99	18.27	65.85	106.07
61	36.74	22.27	39.29	15.24	57.90	101.15
62	24.91	24.58	49.02	13.50	64.38	128.57
63	35.19	20.72	34.27	13.81	51.24	83.67
64	33.94	23.42	41.14	15.21	59.84	104.87
65	39.14	21.06	35.10	14.01	51.48	85.06
66	31.15	20.29	33.20	13.55	50.94	81.87
67	32.94	23.83	41.94	15.97	60.33	105.93
68	28.80	23.05	40.56	16.61	55.26	97.36
69	34.88	23.93	41.43	16.11	58.92	101.49
70	33.38	22.43	38.45	15.25	55.39	94.34
71	38.93	26.10	41.99	18.27	65.85	106.07
72	36.74	22.27	39.29	15.24	57.90	101.15

	VSL	STR	LIN	WOB	ALH	BCF
37	36.38	.66	.38	.57	4.47	26.93
38	33.48	.63	.34	.54	4.43	23.93
39	34.62	.66	.37	.57	4.26	26.03
40	36.21	.68	.39	.57	4.53	26.75
41	39.11	.69	.40	.57	4.21	26.98
42	36.80	.64	.36	.57	4.85	28.08
43	36.69	.67	.38	.57	4.46	26.96
44	34.50	.67	.41	.61	4.43	24.56
45	34.60	.67	.40	.60	4.23	25.46
46	34.37	.67	.41	.62	4.68	23.41
47	40.54	.67	.38	.56	4.44	27.34
48	36.00	.67	.40	.60	4.45	25.19
49	39.69	.71	.40	.56	3.80	30.89
50	40.28	.68	.39	.58	4.17	29.97
51	45.98	.69	.43	.62	4.21	26.90
52	34.50	.67	.41	.61	4.43	24.56
53	40.11	.69	.41	.59	4.15	28.08
54	34.60	.67	.40	.60	4.23	25.46
55	34.37	.67	.41	.62	4.68	23.41
56	40.54	.67	.38	.56	4.44	27.34
57	39.69	.71	.40	.56	3.80	30.89
58	40.28	.68	.39	.58	4.17	29.97
59	37.90	.68	.40	.58	4.26	27.41
60	45.98	.69	.43	.62	4.21	26.90
61	39.82	.68	.39	.57	4.69	25.60
62	35.83	.55	.27	.50	5.21	25.01
63	34.50	.67	.41	.61	4.43	24.56
64	39.03	.65	.38	.58	4.64	25.52
65	34.60	.67	.40	.60	4.23	25.46
66	34.37	.67	.41	.62	4.68	23.41
67	40.54	.67	.38	.56	4.44	27.34
68	39.69	.71	.40	.56	3.80	30.89
69	40.28	.68	.39	.58	4.17	29.97
70	37.90	.68	.40	.58	4.26	27.41
71	45.98	.69	.43	.62	4.21	26.90
72	39.82	.68	.39	.57	4.69	25.60

	Pool	field	Interaction	Group	DMA	TM
73	6	3	LC6	LC	6	40.70
74	6	4	LC6	LC	6	52.09
75	6	5	LC6	LC	6	50.01
76	7	1	LC6	LC	6	57.44
77	7	2	LC6	LC	6	46.62
78	7	3	LC6	LC	6	49.01
79	7	4	LC6	LC	6	43.20
80	7	5	LC6	LC	6	49.07
81	8	1	LC6	LC	6	49.76
82	8	2	LC6	LC	6	47.95
83	8	3	LC6	LC	6	59.31
84	8	4	LC6	LC	6	40.70
85	8	5	LC6	LC	6	49.43
86	1	1	LC9	LC	9	43.73
87	1	2	LC9	LC	9	52.68
88	1	3	LC9	LC	9	37.76
89	1	4	LC9	LC	9	45.24
90	1	5	LC9	LC	9	44.85
91	2	1	LC9	LC	9	38.48
92	2	2	LC9	LC	9	43.73
93	2	3	LC9	LC	9	52.68
94	2	4	LC9	LC	9	37.76
95	2	5	LC9	LC	9	43.16
96	3	1	LC9	LC	9	45.24
97	3	2	LC9	LC	9	38.48
98	3	3	LC9	LC	9	42.93
99	3	4	LC9	LC	9	44.07
100	3	5	LC9	LC	9	42.68
101	4	1	LC9	LC	9	39.04
102	4	2	LC9	LC	9	46.66
103	4	3	LC9	LC	9	41.53
104	4	4	LC9	LC	9	43.73
105	4	5	LC9	LC	9	52.68
106	5	1	LC9	LC	9	37.76
107	5	2	LC9	LC	9	45.24
108	5	3	LC9	LC	9	38.48

CASA-DATA2.sav

	Prog	DAP	DCL	DSL	VAP	VCL
73	24.91	24.58	49.02	13.50	64.38	128.57
74	35.19	20.72	34.27	13.81	51.24	83.67
75	33.94	23.42	41.14	15.21	59.84	104.87
76	39.14	21.06	35.10	14.01	51.48	85.06
77	31.15	20.29	33.20	13.55	50.94	81.87
78	32.94	23.83	41.94	15.97	60.33	105.93
79	28.80	23.05	40.56	16.61	55.26	97.36
80	33.01	22.06	37.70	15.04	54.50	92.56
81	34.88	23.93	41.43	16.11	58.92	101.49
82	38.93	26.10	41.99	18.27	65.85	106.07
83	36.74	22.27	39.29	15.24	57.90	101.15
84	24.91	24.58	49.02	13.50	64.38	128.57
85	33.87	24.22	42.93	15.78	61.76	109.32
86	26.27	19.71	35.33	12.64	49.79	88.25
87	35.33	20.58	37.97	12.33	53.17	96.88
88	21.32	19.27	33.55	13.53	46.47	79.96
89	26.23	18.17	31.95	11.46	46.00	80.89
90	27.29	19.43	34.70	12.49	48.86	86.50
91	21.30	20.29	36.06	13.62	51.20	88.98
92	26.27	19.71	35.33	12.64	49.79	88.25
93	35.33	20.58	37.97	12.33	53.17	96.88
94	21.32	19.27	33.55	13.53	46.47	79.96
95	26.06	19.96	35.73	13.03	50.16	88.52
96	26.23	18.17	31.95	11.46	46.00	80.89
97	21.30	20.29	36.06	13.62	51.20	88.98
98	24.31	21.12	35.63	13.86	52.01	87.58
99	26.06	24.15	39.51	16.03	59.05	96.73
100	24.48	20.93	35.79	13.74	52.07	88.55
101	21.51	20.34	33.14	14.74	49.38	81.12
102	28.25	21.88	36.46	13.65	55.19	91.57
103	21.40	18.25	33.34	11.65	44.14	79.96
104	26.27	19.71	35.33	12.64	49.79	88.25
105	35.33	20.58	37.97	12.33	53.17	96.88
106	21.32	19.27	33.55	13.53	46.47	79.96
107	26.23	18.17	31.95	11.46	46.00	80.89
108	21.30	20.29	36.06	13.62	51.20	88.98

	VSL	STR	LIN	WOB	ALH	BCF
73	35.83	.55	.27	.50	5.21	25.01
74	34.50	.67	.41	.61	4.43	24.56
75	39.03	.65	.38	.58	4.64	25.52
76	34.60	.67	.40	.60	4.23	25.46
77	34.37	.67	.41	.62	4.68	23.41
78	40.54	.67	.38	.56	4.44	27.34
79	39.69	.71	.40	.56	3.80	30.89
80	37.30	.68	.40	.59	4.29	26.78
81	40.28	.68	.39	.58	4.17	29.97
82	45.98	.69	.43	.62	4.21	26.90
83	39.82	.68	.39	.57	4.69	25.60
84	35.83	.55	.27	.50	5.21	25.01
85	40.48	.65	.37	.57	4.57	26.87
86	32.36	.64	.36	.56	4.42	23.94
87	32.27	.60	.33	.54	4.49	24.56
88	32.94	.70	.41	.58	4.32	22.86
89	29.39	.63	.36	.56	3.92	26.47
90	31.74	.64	.37	.56	4.29	24.46
91	35.26	.68	.39	.57	4.94	21.07
92	32.36	.64	.36	.56	4.42	23.94
93	32.27	.60	.33	.54	4.49	24.56
94	32.94	.70	.41	.58	4.32	22.86
95	33.21	.66	.37	.56	4.54	23.11
96	29.39	.63	.36	.56	3.92	26.47
97	35.26	.68	.39	.57	4.94	21.07
98	34.25	.65	.39	.59	4.36	25.78
99	39.09	.66	.40	.61	4.61	28.63
100	34.50	.66	.39	.58	4.46	25.49
101	35.52	.71	.43	.60	4.00	23.62
102	34.79	.63	.37	.60	4.59	24.33
103	28.55	.64	.35	.55	4.14	27.08
104	32.36	.64	.36	.56	4.42	23.94
105	32.27	.60	.33	.54	4.49	24.56
106	32.94	.70	.41	.58	4.32	22.86
107	29.39	.63	.36	.56	3.92	26.47
108	35.26	.68	.39	.57	4.94	21.07

	Pool	field	Interaction	Group	DMA	TM
109	5	4	LC9	LC	9	42.93
110	5	5	LC9	LC	9	44.07
111	5	6	LC9	LC	9	41.70
112	6	1	LC9	LC	9	39.04
113	6	2	LC9	LC	9	46.66
114	6	3	LC9	LC	9	41.53
115	6	4	LC9	LC	9	43.73
116	6	5	LC9	LC	9	42.74
117	7	1	LC9	LC	9	52.68
118	7	2	LC9	LC	9	37.76
119	7	3	LC9	LC	9	45.24
120	7	4	LC9	LC	9	38.48
121	7	5	LC9	LC	9	43.54
122	8	1	LC9	LC	9	42.93
123	8	2	LC9	LC	9	44.07
124	8	3	LC9	LC	9	39.04
125	8	4	LC9	LC	9	46.66
126	8	5	LC9	LC	9	41.53
127	1	1	HC3	HC	3	29.77
128	1	2	HC3	HC	3	30.40
129	1	3	HC3	HC	3	25.00
130	1	4	HC3	HC	3	29.77
131	1	5	HC3	HC	3	30.40
132	1	6	HC3	HC	3	25.00
133	2	1	HC3	HC	3	30.20
134	2	2	HC3	HC	3	30.64
135	2	3	HC3	HC	3	26.53
136	2	4	HC3	HC	3	30.20
137	2	5	HC3	HC	3	30.64
138	2	6	HC3	HC	3	26.53
139	3	1	HC3	HC	3	29.50
140	3	2	HC3	HC	3	40.27
141	3	3	HC3	HC	3	70.82
142	3	4	HC3	HC	3	29.50
143	3	5	HC3	HC	3	40.27
144	3	6	HC3	HC	3	70.82

	Prog	DAP	DCL	DSL	VAP	VCL
109	24.31	21.12	35.63	13.86	52.01	87.58
110	26.06	24.15	39.51	16.03	59.05	96.73
111	23.84	20.60	35.34	13.70	50.95	86.83
112	21.51	20.34	33.14	14.74	49.38	81.12
113	28.25	21.88	36.46	13.65	55.19	91.57
114	21.40	18.25	33.34	11.65	44.14	79.96
115	26.27	19.71	35.33	12.64	49.79	88.25
116	24.36	20.05	34.57	13.17	49.63	85.23
117	35.33	20.58	37.97	12.33	53.17	96.88
118	21.32	19.27	33.55	13.53	46.47	79.96
119	26.23	18.17	31.95	11.46	46.00	80.89
120	21.30	20.29	36.06	13.62	51.20	88.98
121	26.05	19.58	34.88	12.74	49.21	86.68
122	24.31	21.12	35.63	13.86	52.01	87.58
123	26.06	24.15	39.51	16.03	59.05	96.73
124	21.51	20.34	33.14	14.74	49.38	81.12
125	28.25	21.88	36.46	13.65	55.19	91.57
126	21.40	18.25	33.34	11.65	44.14	79.96
127	10.43	25.25	49.65	14.36	64.04	125.85
128	10.40	25.95	54.04	13.45	57.64	120.25
129	11.53	21.71	41.55	12.00	53.64	101.65
130	10.43	25.25	49.65	14.36	64.04	125.85
131	10.40	25.95	54.04	13.45	57.64	120.25
132	11.53	21.71	41.55	12.00	53.64	101.65
133	14.76	24.75	49.44	17.02	72.97	147.11
134	12.90	24.55	47.74	13.13	60.16	116.28
135	8.84	24.63	48.59	14.81	57.96	113.39
136	14.76	24.75	49.44	17.02	72.97	147.11
137	12.90	24.55	47.74	13.13	60.16	116.28
138	8.84	24.63	48.59	14.81	57.96	113.39
139	3.27	22.48	36.89	13.84	59.27	97.01
140	11.11	34.63	67.17	14.09	85.55	166.64
141	37.63	24.95	48.58	14.37	59.84	116.44
142	3.27	22.48	36.89	13.84	59.27	97.01
143	11.11	34.63	67.17	14.09	85.55	166.64
144	37.63	24.95	48.58	14.37	59.84	116.44

	VSL	STR	LIN	WOB	ALH	BCF
109	34.25	.65	.39	.59	4.36	25.78
110	39.09	.66	.40	.61	4.61	28.63
111	34.19	.66	.39	.58	4.43	24.96
112	35.52	.71	.43	.60	4.00	23.62
113	34.79	.63	.37	.60	4.59	24.33
114	28.55	.64	.35	.55	4.14	27.08
115	32.36	.64	.36	.56	4.42	23.94
116	32.81	.66	.38	.58	4.29	24.74
117	32.27	.60	.33	.54	4.49	24.56
118	32.94	.70	.41	.58	4.32	22.86
119	29.39	.63	.36	.56	3.92	26.47
120	35.26	.68	.39	.57	4.94	21.07
121	32.47	.65	.37	.56	4.42	23.74
122	34.25	.65	.39	.59	4.36	25.78
123	39.09	.66	.40	.61	4.61	28.63
124	35.52	.71	.43	.60	4.00	23.62
125	34.79	.63	.37	.60	4.59	24.33
126	28.55	.64	.35	.55	4.14	27.08
127	36.76	.57	.29	.50	5.54	26.88
128	29.99	.52	.24	.47	4.64	27.00
129	29.93	.55	.29	.52	4.94	25.32
130	36.76	.57	.29	.50	5.54	26.88
131	29.99	.52	.24	.47	4.64	27.00
132	29.93	.55	.29	.52	4.94	25.32
133	50.03	.68	.34	.49	5.95	30.01
134	31.91	.53	.27	.51	5.86	26.07
135	34.55	.59	.30	.51	5.24	25.50
136	50.03	.68	.34	.49	5.95	30.01
137	31.91	.53	.27	.51	5.86	26.07
138	34.55	.59	.30	.51	5.24	25.50
139	37.19	.62	.38	.61	4.82	15.24
140	34.65	.40	.20	.51	6.95	30.14
141	34.52	.57	.29	.51	5.58	26.74
142	37.19	.62	.38	.61	4.82	15.24
143	34.65	.40	.20	.51	6.95	30.14
144	34.52	.57	.29	.51	5.58	26.74

	Pool	field	Interaction	Group	DMA	TM
145	4	1	HC3	HC	3	70.17
146	4	2	HC3	HC	3	72.09
147	4	3	HC3	HC	3	68.18
148	4	4	HC3	HC	3	70.17
149	4	5	HC3	HC	3	72.09
150	4	6	HC3	HC	3	68.18
151	5	1	HC3	HC	3	81.33
152	5	2	HC3	HC	3	67.34
153	5	3	HC3	HC	3	65.15
154	5	4	HC3	HC	3	81.33
155	5	5	HC3	HC	3	67.34
156	5	6	HC3	HC	3	65.15
157	6	1	HC3	HC	3	60.43
158	6	2	HC3	HC	3	60.65
159	6	3	HC3	HC	3	74.02
160	6	4	HC3	HC	3	60.65
161	?	?	?	?	?	?
162	?	?	?	?	?	?
163	?	?	?	?	?	?
164	?	?	?	?	?	?
165	?	?	?	?	?	?
166	?	?	?	?	?	?
167	?	?	?	?	?	?
168	?	?	?	?	?	?
169	?	?	?	?	?	?
170	?	?	?	?	?	?
171	?	?	?	?	?	?
172	?	?	?	?	?	?
173	?	?	?	?	?	?
174	?	?	?	?	?	?
175	?	?	?	?	?	?
176	?	?	?	?	?	?
177	?	?	?	?	?	?
178	?	?	?	?	?	?
179	?	?	?	?	?	?
180	?	?	?	?	?	?

CASA-DATA2.sav

	Prog	DAP	DCL	DSL	VAP	VCL
145	28.07	22.71	38.55	15.13	53.87	90.98
146	20.93	20.50	49.96	10.88	44.65	108.46
147	43.75	26.70	51.45	15.35	66.52	128.79
148	28.07	22.71	38.55	15.13	53.87	90.98
149	20.93	20.50	49.96	10.88	44.65	108.46
150	43.75	26.70	51.45	15.35	66.52	128.79
151	38.66	24.93	50.76	14.13	55.73	113.11
152	38.77	23.43	45.00	13.09	55.58	106.58
153	36.43	32.36	72.65	15.41	82.65	185.35
154	38.66	24.93	50.76	14.13	55.73	113.11
155	38.77	23.43	45.00	13.09	55.58	106.58
156	36.43	32.36	72.65	15.41	82.65	185.35
157	38.46	35.72	83.66	14.91	87.63	204.29
158	36.06	33.12	79.18	16.01	86.28	206.26
159	36.36	26.24	50.66	14.59	71.03	139.59
160	36.06	33.12	79.18	16.01	86.28	206.26
161	36.36	26.24	50.66	14.59	71.03	139.59
162	34.88	33.04	70.75	15.88	82.37	175.32
163	26.46	23.86	49.91	12.76	59.01	123.49
164	26.51	21.49	42.99	13.68	51.14	101.79
165	34.88	33.04	70.75	15.88	82.37	175.32
166	26.46	23.86	49.91	12.76	59.01	123.49
167	30.11	26.09	51.22	12.44	65.01	127.98
168	22.67	23.76	56.65	12.05	60.54	144.08
169	30.11	26.09	51.22	12.44	65.01	127.98
170	22.67	23.76	56.65	12.05	60.54	144.08
171	22.67	23.76	56.65	12.05	60.54	144.08
172	30.11	26.09	51.22	12.44	65.01	127.98
173	30.42	27.09	50.33	14.65	67.48	124.77
174	39.83	26.61	48.07	12.90	65.88	119.04
175	22.85	17.72	34.16	10.95	43.95	82.46
176	30.42	27.09	50.33	14.65	67.48	124.77
177	39.83	26.61	48.07	12.90	65.88	119.04
178	22.85	17.72	34.16	10.95	43.95	82.46
179	29.78	29.71	58.64	15.49	74.33	145.20
180	18.75	24.57	40.07	16.51	63.22	101.16

	VSL	STR	LIN	WOB	ALH	BCF
145	36.15	.67	.39	.59	5.08	32.81
146	23.88	.53	.22	.41	5.54	22.75
147	38.01	.57	.29	.51	5.92	26.17
148	36.15	.67	.39	.59	5.08	32.81
149	23.88	.53	.22	.41	5.54	22.75
150	38.01	.57	.29	.51	5.92	26.17
151	31.69	.56	.28	.49	5.38	26.52
152	31.41	.56	.29	.52	5.27	26.48
153	39.31	.47	.21	.44	6.00	32.13
154	31.69	.56	.28	.49	5.38	26.52
155	31.41	.56	.29	.52	5.27	26.48
156	39.31	.47	.21	.44	6.00	32.13
157	36.77	.41	.17	.42	6.49	30.91
158	41.59	.48	.20	.41	6.02	36.78
159	38.45	.54	.27	.50	5.22	27.96
160	41.59	.48	.20	.41	6.02	36.78
161	38.45	.54	.27	.50	5.22	27.96
162	39.74	.48	.22	.46	6.11	30.63
163	31.88	.54	.25	.47	5.30	27.32
164	32.78	.64	.32	.50	5.31	26.34
165	39.74	.48	.22	.46	6.11	30.63
166	31.88	.54	.25	.47	5.30	27.32
167	31.44	.48	.24	.50	5.05	25.19
168	31.36	.51	.21	.42	5.62	31.42
169	31.44	.48	.24	.50	5.05	25.19
170	31.36	.51	.21	.42	5.62	31.42
171	31.36	.51	.21	.42	5.62	31.42
172	31.44	.48	.24	.50	5.05	25.19
173	37.18	.55	.29	.54	5.64	28.36
174	32.08	.48	.26	.55	5.75	28.50
175	27.39	.62	.33	.53	4.46	35.09
176	37.18	.55	.29	.54	5.64	28.36
177	32.08	.48	.26	.55	5.75	28.50
178	27.39	.62	.33	.53	4.46	35.09
179	39.82	.53	.27	.51	6.13	26.38
180	44.14	.69	.43	.62	5.17	27.32

	Pool	field	Interaction	Group	DMA	TM
181	2	4	HC6	HC	6	40.47
182	2	5	HC6	HC	6	52.65
183	3	1	HC6	HC	6	70.07
184	3	2	HC6	HC	6	73.24
185	3	3	HC6	HC	6	71.79
186	3	4	HC6	HC	6	70.07
187	3	5	HC6	HC	6	73.24
188	3	6	HC6	HC	6	71.79
189	4	1	HC6	HC	6	90.90
190	4	2	HC6	HC	6	82.05
191	4	3	HC6	HC	6	65.42
192	4	4	HC6	HC	6	82.05
193	4	5	HC6	HC	6	65.42
194	5	1	HC6	HC	6	73.43
195	5	2	HC6	HC	6	55.26
196	5	3	HC6	HC	6	54.11
197	5	4	HC6	HC	6	55.26
198	5	5	HC6	HC	6	54.11
199	6	1	HC6	HC	6	46.93
200	6	2	HC6	HC	6	52.17
201	6	3	HC6	HC	6	61.90
202	6	4	HC6	HC	6	52.17
203	6	5	HC6	HC	6	61.90
204	7	1	HC6	HC	6	61.85
205	7	2	HC6	HC	6	57.95
206	7	3	HC6	HC	6	57.56
207	7	4	HC6	HC	6	61.85
208	7	5	HC6	HC	6	57.95
209	8	1	HC6	HC	6	49.26
210	8	2	HC6	HC	6	57.73
211	8	3	HC6	HC	6	70.06
212	8	4	HC6	HC	6	49.26
213	8	5	HC6	HC	6	57.73
214	8	6	HC6	HC	6	70.06
215	1	1	HC9	HC	9	34.08
216	1	2	HC9	HC	9	30.33

	Prog	DAP	DCL	DSL	VAP	VCL
181	25.00	25.36	48.12	12.83	61.55	117.55
182	29.78	29.71	58.64	15.49	74.33	145.20
183	45.98	29.72	54.62	16.57	74.33	136.58
184	43.14	26.15	49.99	13.97	63.33	120.65
185	35.89	19.54	32.62	10.66	48.40	78.99
186	45.98	29.72	54.62	16.57	74.33	136.58
187	43.14	26.15	49.99	13.97	63.33	120.65
188	35.89	19.54	32.62	10.66	48.40	78.99
189	27.27	33.61	62.73	24.68	105.90	191.97
190	47.43	26.84	52.54	14.71	62.28	121.95
191	42.05	27.73	53.07	14.19	68.74	131.82
192	47.43	26.84	52.54	14.71	62.28	121.95
193	42.05	27.73	53.07	14.19	68.74	131.82
194	46.87	25.28	49.05	13.20	59.25	114.61
195	33.16	27.98	56.30	14.97	75.20	150.60
196	25.88	30.03	57.89	18.95	84.47	162.49
197	33.16	27.98	56.30	14.97	75.20	150.60
198	25.88	30.03	57.89	18.95	84.47	162.49
199	32.65	27.98	58.68	13.65	74.75	156.26
200	26.08	23.18	47.23	10.21	64.45	131.86
201	36.50	29.52	62.04	13.63	77.57	161.16
202	26.08	23.18	47.23	10.21	64.45	131.86
203	36.50	29.52	62.04	13.63	77.57	161.16
204	41.23	27.42	52.92	16.05	72.32	139.09
205	28.97	22.30	46.52	13.07	55.63	115.63
206	31.21	21.18	47.82	11.95	52.28	116.54
207	41.23	27.42	52.92	16.05	72.32	139.09
208	28.97	22.30	46.52	13.07	55.63	115.63
209	25.36	24.34	50.42	14.10	60.47	126.26
210	27.97	24.64	52.28	13.24	62.63	132.00
211	31.84	19.42	35.39	13.27	48.31	88.03
212	25.36	24.34	50.42	14.10	60.47	126.26
213	27.97	24.64	52.28	13.24	62.63	132.00
214	31.84	19.42	35.39	13.27	48.31	88.03
215	16.33	21.69	41.14	13.41	54.78	104.14
216	15.73	23.45	41.37	15.63	54.08	94.53

	VSL	STR	LIN	WOB	ALH	BCF
181	31.10	.50	.26	.52	5.19	30.91
182	39.82	.53	.27	.51	6.13	26.38
183	42.18	.56	.30	.54	5.90	27.04
184	34.17	.53	.28	.52	5.46	26.17
185	26.36	.54	.33	.61	4.64	19.13
186	42.18	.56	.30	.54	5.90	27.04
187	34.17	.53	.28	.52	5.46	26.17
188	26.36	.54	.33	.61	4.64	19.13
189	78.95	.74	.41	.55	8.21	35.93
190	34.18	.54	.28	.51	5.84	27.87
191	35.80	.52	.27	.52	5.44	27.48
192	34.18	.54	.28	.51	5.84	27.87
193	35.80	.52	.27	.52	5.44	27.48
194	30.88	.52	.26	.51	5.12	24.44
195	40.97	.54	.27	.49	6.03	31.01
196	54.21	.64	.33	.51	6.75	31.72
197	40.97	.54	.27	.49	6.03	31.01
198	54.21	.64	.33	.51	6.75	31.72
199	37.20	.49	.23	.47	6.34	31.10
200	29.45	.45	.22	.48	4.45	30.78
201	35.83	.46	.22	.48	5.53	31.30
202	29.45	.45	.22	.48	4.45	30.78
203	35.83	.46	.22	.48	5.53	31.30
204	43.12	.59	.31	.51	6.16	30.45
205	32.92	.59	.28	.48	4.90	25.72
206	29.92	.57	.25	.44	5.22	24.22
207	43.12	.59	.31	.51	6.16	30.45
208	32.92	.59	.28	.48	4.90	25.72
209	35.40	.58	.28	.47	5.22	27.21
210	33.90	.54	.25	.47	4.61	26.86
211	33.26	.68	.37	.54	4.42	25.01
212	35.40	.58	.28	.47	5.22	27.21
213	33.90	.54	.25	.47	4.61	26.86
214	33.26	.68	.37	.54	4.42	25.01
215	34.45	.62	.33	.52	4.79	26.43
216	36.42	.67	.38	.57	4.39	29.38

	Pool	field	Interaction	Group	DMA	TM
217	1	3	HC9	HC	9	34.14
218	1	4	HC9	HC	9	34.08
219	1	5	HC9	HC	9	30.33
220	1	6	HC9	HC	9	32.59
221	2	1	HC9	HC	9	29.62
222	2	2	HC9	HC	9	34.65
223	2	3	HC9	HC	9	32.18
224	2	4	HC9	HC	9	29.62
225	2	5	HC9	HC	9	34.65
226	2	6	HC9	HC	9	32.14
227	3	1	HC9	HC	9	37.45
228	3	2	HC9	HC	9	53.38
229	3	3	HC9	HC	9	55.55
230	3	4	HC9	HC	9	37.45
231	3	5	HC9	HC	9	53.38
232	3	6	HC9	HC	9	47.44
233	4	1	HC9	HC	9	50.00
234	4	2	HC9	HC	9	55.17
235	4	3	HC9	HC	9	58.80
236	4	4	HC9	HC	9	50.00
237	4	5	HC9	HC	9	55.17
238	4	6	HC9	HC	9	53.83
239	5	1	HC9	HC	9	61.51
240	5	2	HC9	HC	9	50.79
241	?	?	?	?	?	?
242	?	?	?	?	?	?
243	?	?	?	?	?	?
244	?	?	?	?	?	?
245	?	?	?	?	?	?
246	?	?	?	?	?	?
247	?	?	?	?	?	?
248	?	?	?	?	?	?
249	?	?	?	?	?	?
250	?	?	?	?	?	?
251	?	?	?	?	?	?
252	?	?	?	?	?	?

	Prog	DAP	DCL	DSL	VAP	VCL
217	14.63	36.94	53.60	23.15	81.13	117.92
218	16.33	21.69	41.14	13.41	54.78	104.14
219	15.73	23.45	41.37	15.63	54.08	94.53
220	15.75	25.44	43.72	16.25	59.77	103.05
221	11.11	25.74	43.68	16.29	?	?
222	11.88	20.38	37.85	10.02	?	?
223	12.64	21.06	34.30	14.78	?	?
224	11.11	25.74	43.68	16.29	?	?
225	11.88	20.38	37.85	10.02	?	?
226	11.72	22.66	39.47	13.48	?	?
227	22.31	19.71	42.87	11.68	?	?
228	18.64	27.48	49.20	16.70	?	?
229	20.00	23.13	38.57	15.01	?	?
230	22.31	19.71	42.87	11.68	?	?
231	18.64	27.48	49.20	16.70	?	?
232	20.38	23.50	44.54	14.35	56.01	107.14
233	20.45	35.05	60.70	20.70	76.96	132.75
234	13.79	20.23	47.26	11.52	43.63	100.63
235	33.62	23.99	47.90	13.68	59.83	119.84
236	20.45	35.05	60.70	20.70	76.96	132.75
237	13.79	20.23	47.26	11.52	43.63	100.63
238	20.42	26.91	52.76	15.62	60.20	117.32
239	36.18	22.48	42.34	13.76	56.42	107.05
240	28.57	29.63	59.47	15.83	71.65	144.45
241	34.18	22.69	47.77	12.53	?	120.58
242	36.18	22.48	42.34	13.76	56.42	107.05
243	28.57	29.63	59.47	15.83	71.65	144.45
244	32.74	25.38	50.28	14.34	62.70	124.72
245	31.26	20.96	39.09	13.36	54.46	100.49
246	33.92	18.84	37.03	12.59	48.73	94.44
247	31.26	20.96	39.09	13.36	54.46	100.49
248	33.92	18.84	37.03	12.59	48.73	94.44
249	32.59	19.90	38.06	12.98	51.60	97.47
250	32.39	18.60	32.98	13.66	48.54	85.58
251	32.40	24.21	44.89	14.01	65.38	119.16
252	32.39	18.60	32.98	13.66	48.54	85.58

	VSL	STR	LIN	WOB	ALH	BCF
217	51.83	.63	.43	.68	4.57	23.72
218	34.45	.62	.33	.52	4.79	26.43
219	36.42	.67	.38	.57	4.39	29.38
220	38.71	.64	.37	.57	4.59	27.07
221	41.51	.63	.36	.58	5.27	23.22
222	23.18	.48	.25	.52	5.92	27.73
223	38.58	.71	.44	.62	4.70	23.11
224	41.51	.63	.36	.58	5.27	23.22
225	23.18	.48	.25	.52	5.92	27.73
226	33.59	.59	.33	.56	5.42	25.00
227	32.13	.60	.28	.46	4.66	27.36
228	37.35	.61	.34	.55	6.59	27.82
229	34.35	.65	.38	.58	7.11	25.97
230	32.13	.60	.28	.46	4.66	27.36
231	37.35	.61	.34	.55	6.59	27.82
232	34.66	.61	.32	.52	5.92	27.27
233	46.06	.59	.34	.57	6.91	31.39
234	24.51	.56	.24	.43	4.70	23.93
235	34.25	.57	.28	.49	5.26	25.95
236	46.06	.59	.34	.57	6.91	31.39
237	24.51	.56	.24	.43	4.70	23.93
238	35.08	.57	.29	.50	5.70	27.32
239	34.30	.60	.32	.52	4.87	25.72
240	38.79	.54	.26	.49	5.96	28.18
241	31.91	.55	.26	.47	5.31	25.07
242	34.30	.60	.32	.52	4.87	25.72
243	38.79	.54	.26	.49	5.96	28.18
244	35.62	.57	.28	.50	5.39	26.57
245	35.12	.64	.34	.54	5.12	23.65
246	33.12	.67	.35	.51	5.13	20.52
247	35.12	.64	.34	.54	5.12	23.65
248	33.12	.67	.35	.51	5.13	20.52
249	34.12	.66	.35	.53	5.13	22.09
250	36.00	.74	.42	.56	4.90	23.80
251	39.06	.59	.32	.54	5.52	26.08
252	36.00	.74	.42	.56	4.90	23.80

CASA-DATA2.sav

	Pool	field	Interaction	Group	DMA	TM
253	7	4	HC9	HC	9	63.12
254	7	5	HC9	HC	9	63.95
255	8	1	HC9	HC	9	66.00
256	8	2	HC9	HC	9	67.42
257	8	3	HC9	HC	9	66.00
258	8	4	HC9	HC	9	67.42
259	8	5	HC9	HC	9	66.71

	Prog	DAP	DCL	DSL	VAP	VCL
253	32.40	24.21	44.89	14.01	65.38	119.16
254	32.40	21.41	38.94	13.84	56.96	102.37
255	32.00	21.61	38.54	14.13	54.47	96.87
256	26.85	20.29	40.14	12.09	51.43	100.65
257	32.00	21.61	38.54	14.13	54.47	96.87
258	26.85	20.29	40.14	12.09	51.43	100.65
259	29.43	20.95	39.34	13.11	52.95	98.76

	VSL	STR	LIN	WOB	ALH	BCF
253	39.06	.59	.32	.54	5.52	26.08
254	37.53	.67	.37	.55	5.21	24.94
255	35.33	.64	.36	.56	5.37	21.75
256	30.83	.59	.30	.51	4.57	24.96
257	35.33	.64	.36	.56	5.37	21.75
258	30.83	.59	.30	.51	4.57	24.96
259	33.08	.62	.33	.54	4.97	23.36
