# Supplementary material for: Effect of semen dilution rate and dimethyl acetamide levels on post-thaw motility and fertility parameters of rooster sperm
Source: PLoS One. 2025 Oct 31;20(10):e0335748. doi: 10.1371/journal.pone.0335748 (PMC12578245; doi:10.1371/journal.pone.0335748)
Supplement: S2 File — (PDF) [file pone.0335748.s002.pdf]

Fertility data1.sav

	Group	DMA	Interaction	Hen	Eggs	Fertile
1	LSC	3	L3	1	1	1
2	LSC	3	L3	1	1	1
3	LSC	3	L3	1	1	1
4	LSC	3	L3	1	1	0
5	LSC	3	L3	1	1	0
6	LSC	3	L3	1	1	0
7	LSC	3	L3	1	1	0
8	LSC	3	L3	1	1	0
9	LSC	3	L3	2	1	0
10	LSC	3	L3	2	1	0
11	LSC	3	L3	2	1	0
12	LSC	3	L3	2	1	0
13	LSC	3	L3	2	1	0
14	LSC	3	L3	2	1	0
15	LSC	3	L3	2	1	0
16	LSC	3	L3	2	1	0
17	LSC	3	L3	3	1	0
18	LSC	3	L3	3	1	0
19	LSC	3	L3	3	1	0
20	LSC	3	L3	3	1	0
21	LSC	3	L3	3	1	0
22	LSC	3	L3	3	1	0
23	LSC	3	L3	3	1	0
24	LSC	3	L3	3	1	0
25	LSC	3	L3	4	1	0
26	LSC	3	L3	4	1	0
27	LSC	3	L3	4	1	0
28	LSC	3	L3	4	1	0
29	LSC	3	L3	4	1	0
30	LSC	3	L3	4	1	0
31	LSC	3	L3	4	1	0
32	LSC	3	L3	4	1	0
33	LSC	3	L3	5	1	0
34	LSC	3	L3	5	1	0
35	LSC	3	L3	5	1	0
36	LSC	3	L3	5	1	0

	Hatched	Pipped	ED	LD	Dead
1	1	0	0	0	0
2	1	0	0	0	0
3	1	0	0	0	0
4	0	0	0	0	0
5	0	0	0	0	0
6	0	0	0	0	0
7	0	0	0	0	0
8	0	0	0	0	0
9	0	0	0	0	0
10	0	0	0	0	0
11	0	0	0	0	0
12	0	0	0	0	0
13	0	0	0	0	0
14	0	0	0	0	0
15	0	0	0	0	0
16	0	0	0	0	0
17	0	0	0	0	0
18	0	0	0	0	0
19	0	0	0	0	0
20	0	0	0	0	0
21	0	0	0	0	0
22	0	0	0	0	0
23	0	0	0	0	0
24	0	0	0	0	0
25	0	0	0	0	0
26	0	0	0	0	0
27	0	0	0	0	0
28	0	0	0	0	0
29	0	0	0	0	0
30	0	0	0	0	0
31	0	0	0	0	0
32	0	0	0	0	0
33	0	0	0	0	0
34	0	0	0	0	0
35	0	0	0	0	0
36	0	0	0	0	0

Fertility data1.sav

	Group	DMA	Interaction	Hen	Eggs	Fertile
37	LSC	3	L3	5	1	0
38	LSC	3	L3	5	1	1
39	LSC	3	L3	5	1	1
40	LSC	3	L3	5	1	1
41	LSC	3	L3	5	1	0
42	LSC	3	L3	6	1	0
43	LSC	3	L3	6	1	0
44	LSC	3	L3	6	1	0
45	LSC	3	L3	6	1	0
46	LSC	3	L3	6	1	0
47	LSC	3	L3	6	1	0
48	LSC	3	L3	6	1	0
49	LSC	3	L3	6	1	0
50	LSC	3	L3	7	1	0
51	LSC	3	L3	7	1	0
52	LSC	3	L3	7	1	0
53	LSC	3	L3	7	1	0
54	LSC	3	L3	7	1	0
55	LSC	3	L3	7	1	0
56	LSC	3	L3	7	1	0
57	LSC	3	L3	7	1	0
58	LSC	3	L3	7	1	0
59	LSC	3	L3	8	1	0
60	LSC	3	L3	8	1	0
61	LSC	3	L3	8	1	0
62	LSC	3	L3	8	1	0
63	LSC	3	L3	8	1	0
64	LSC	3	L3	8	1	0
65	LSC	3	L3	8	1	0
66	LSC	3	L3	8	1	0
67	LSC	3	L3	8	1	0
68	LSC	3	L3	9	1	0
69	LSC	3	L3	9	1	0
70	LSC	3	L3	9	1	0
71	LSC	3	L3	9	1	0
72	LSC	3	L3	9	1	0

Fertility data1.sav

	Hatched	Pipped	ED	LD	Dead
37	0	0	0	0	0
38	1	0	0	0	0
39	1	0	0	0	0
40	1	0	0	0	0
41	0	0	0	0	0
42	0	0	0	0	0
43	0	0	0	0	0
44	0	0	0	0	0
45	0	0	0	0	0
46	0	0	0	0	0
47	0	0	0	0	0
48	0	0	0	0	0
49	0	0	0	0	0
50	0	0	0	0	0
51	0	0	0	0	0
52	0	0	0	0	0
53	0	0	0	0	0
54	0	0	0	0	0
55	0	0	0	0	0
56	0	0	0	0	0
57	0	0	0	0	0
58	0	0	0	0	0
59	0	0	0	0	0
60	0	0	0	0	0
61	0	0	0	0	0
62	0	0	0	0	0
63	0	0	0	0	0
64	0	0	0	0	0
65	0	0	0	0	0
66	0	0	0	0	0
67	0	0	0	0	0
68	0	0	0	0	0
69	0	0	0	0	0
70	0	0	0	0	0
71	0	0	0	0	0
72	0	0	0	0	0

Fertility data1.sav

	Group	DMA	Interaction	Hen	Eggs	Fertile
73	LSC	3	L3	9	1	0
74	LSC	3	L3	9	1	0
75	LSC	3	L3	9	1	0
76	LSC	3	L3	9	1	0
77	LSC	3	L3	10	1	0
78	LSC	3	L3	10	1	0
79	LSC	3	L3	10	1	0
80	LSC	3	L3	10	1	0
81	LSC	3	L3	10	1	0
82	LSC	3	L3	10	1	0
83	LSC	3	L3	10	1	0
84	LSC	3	L3	10	1	0
85	LSC	3	L3	10	1	0
86	LSC	6	L6	11	1	0
87	LSC	6	L6	11	1	0
88	LSC	6	L6	11	1	0
89	LSC	6	L6	11	1	0
90	LSC	6	L6	11	1	0
91	LSC	6	L6	11	1	0
92	LSC	6	L6	11	1	0
93	LSC	6	L6	11	1	0
94	LSC	6	L6	11	1	0
95	LSC	6	L6	12	1	0
96	LSC	6	L6	12	1	0
97	LSC	6	L6	12	1	0
98	LSC	6	L6	12	1	0
99	LSC	6	L6	12	1	0
100	LSC	6	L6	12	1	0
101	LSC	6	L6	12	1	0
102	LSC	6	L6	12	1	0
103	LSC	6	L6	12	1	0
104	LSC	6	L6	13	1	0
105	LSC	6	L6	13	1	0
106	LSC	6	L6	13	1	0
107	LSC	6	L6	13	1	0
108	LSC	6	L6	13	1	0

...	Hatched	Pipped	ED	LD	Dead
73	0	0	0	0	0
74	0	0	0	0	0
75	0	0	0	0	0
76	0	0	0	0	0
77	0	0	0	0	0
78	0	0	0	0	0
79	0	0	0	0	0
80	0	0	0	0	0
81	0	0	0	0	0
82	0	0	0	0	0
83	0	0	0	0	0
84	0	0	0	0	0
85	0	0	0	0	0
86	0	0	0	0	0
87	0	0	0	0	0
88	0	0	0	0	0
89	0	0	0	0	0
90	0	0	0	0	0
91	0	0	0	0	0
92	0	0	0	0	0
93	0	0	0	0	0
94	0	0	0	0	0
95	0	0	0	0	0
96	0	0	0	0	0
97	0	0	0	0	0
98	0	0	0	0	0
99	0	0	0	0	0
100	0	0	0	0	0
101	0	0	0	0	0
102	0	0	0	0	0
103	0	0	0	0	0
104	0	0	0	0	0
105	0	0	0	0	0
106	0	0	0	0	0
107	0	0	0	0	0
108	0	0	0	0	0

Fertility data1.sav

	Group	DMA	Interaction	Hen	Eggs	Fertile
109	LSC	6	L6	13	1	0
110	LSC	6	L6	13	1	0
111	LSC	6	L6	13	1	0
112	LSC	6	L6	13	1	0
113	LSC	6	L6	14	1	0
114	LSC	6	L6	14	1	0
115	LSC	6	L6	14	1	1
116	LSC	6	L6	14	1	1
117	LSC	6	L6	14	1	1
118	LSC	6	L6	14	1	0
119	LSC	6	L6	14	1	0
120	LSC	6	L6	15	1	0
121	LSC	6	L6	15	1	0
122	LSC	6	L6	15	1	0
123	LSC	6	L6	15	1	0
124	LSC	6	L6	15	1	0
125	LSC	6	L6	15	1	0
126	LSC	6	L6	15	1	0
127	LSC	6	L6	15	1	0
128	LSC	6	L6	16	1	0
129	LSC	6	L6	16	1	0
130	LSC	6	L6	16	1	0
131	LSC	6	L6	16	1	0
132	LSC	6	L6	16	1	0
133	LSC	6	L6	16	1	0
134	LSC	6	L6	16	1	0
135	LSC	6	L6	16	1	0
136	LSC	6	L6	16	1	0
137	LSC	6	L6	17	1	0
138	LSC	6	L6	17	1	0
139	LSC	6	L6	17	1	1
140	LSC	6	L6	17	1	0
141	LSC	6	L6	17	1	0
142	LSC	6	L6	17	1	0
143	LSC	6	L6	17	1	0
144	LSC	6	L6	17	1	0

Fertility data1.sav

	Hatched	Pipped	ED	LD	Dead
109	0	0	0	0	0
110	0	0	0	0	0
111	0	0	0	0	0
112	0	0	0	0	0
113	0	0	0	0	0
114	0	0	0	0	0
115	1	0	0	0	0
116	1	0	0	0	0
117	1	0	0	0	0
118	0	0	0	0	0
119	0	0	0	0	0
120	0	0	0	0	0
121	0	0	0	0	0
122	0	0	0	0	0
123	0	0	0	0	0
124	0	0	0	0	0
125	0	0	0	0	0
126	0	0	0	0	0
127	0	0	0	0	0
128	0	0	0	0	0
129	0	0	0	0	0
130	0	0	0	0	0
131	0	0	0	0	0
132	0	0	0	0	0
133	0	0	0	0	0
134	0	0	0	0	0
135	0	0	0	0	0
136	0	0	0	0	0
137	0	0	0	0	0
138	0	0	0	0	0
139	1	0	0	0	0
140	0	0	0	0	0
141	0	0	0	0	0
142	0	0	0	0	0
143	0	0	0	0	0
144	0	0	0	0	0

Fertility data1.sav

	Group	DMA	Interaction	Hen	Eggs	Fertile
145	LSC	6	L6	18	1	0
146	LSC	6	L6	18	1	0
147	LSC	6	L6	18	1	0
148	LSC	6	L6	18	1	0
149	LSC	6	L6	18	1	0
150	LSC	6	L6	18	1	0
151	LSC	6	L6	18	1	0
152	LSC	6	L6	18	1	0
153	LSC	6	L6	19	1	0
154	LSC	6	L6	19	1	0
155	LSC	6	L6	19	1	0
156	LSC	6	L6	19	1	0
157	LSC	6	L6	19	1	0
158	LSC	6	L6	19	1	0
159	LSC	6	L6	19	1	0
160	LSC	6	L6	19	1	0
161	LSC	6	L6	19	1	1
162	LSC	6	L6	20	1	0
163	LSC	6	L6	20	1	0
164	LSC	6	L6	20	1	0
165	LSC	6	L6	20	1	0
166	LSC	6	L6	20	1	0
167	LSC	6	L6	20	1	0
168	LSC	6	L6	20	1	0
169	LSC	6	L6	20	1	0
170	LSC	9	L9	21	1	0
171	LSC	9	L9	21	1	0
172	LSC	9	L9	21	1	0
173	LSC	9	L9	21	1	0
174	LSC	9	L9	21	1	0
175	LSC	9	L9	21	1	0
176	LSC	9	L9	21	1	0
177	LSC	9	L9	21	1	0
178	LSC	9	L9	21	1	0
179	LSC	9	L9	22	1	0
180	LSC	9	L9	22	1	0

Fertility data1.sav

	Hatched	Pipped	ED	LD	Dead
145	0	0	0	0	0
146	0	0	0	0	0
147	0	0	0	0	0
148	0	0	0	0	0
149	0	0	0	0	0
150	0	0	0	0	0
151	0	0	0	0	0
152	0	0	0	0	0
153	0	0	0	0	0
154	0	0	0	0	0
155	0	0	0	0	0
156	0	0	0	0	0
157	0	0	0	0	0
158	0	0	0	0	0
159	0	0	0	0	0
160	0	0	0	0	0
161	1	0	0	0	0
162	0	0	0	0	0
163	0	0	0	0	0
164	0	0	0	0	0
165	0	0	0	0	0
166	0	0	0	0	0
167	0	0	0	0	0
168	0	0	0	0	0
169	0	0	0	0	0
170	0	0	0	0	0
171	0	0	0	0	0
172	0	0	0	0	0
173	0	0	0	0	0
174	0	0	0	0	0
175	0	0	0	0	0
176	0	0	0	0	0
177	0	0	0	0	0
178	0	0	0	0	0
179	0	0	0	0	0
180	0	0	0	0	0

Fertility data1.sav

	Group	DMA	Interaction	Hen	Eggs	Fertile
181	LSC	9	L9	22	1	0
182	LSC	9	L9	22	1	0
183	LSC	9	L9	22	1	0
184	LSC	9	L9	22	1	0
185	LSC	9	L9	22	1	0
186	LSC	9	L9	22	1	0
187	LSC	9	L9	23	1	0
188	LSC	9	L9	23	1	0
189	LSC	9	L9	23	1	0
190	LSC	9	L9	23	1	0
191	LSC	9	L9	23	1	0
192	LSC	9	L9	23	1	0
193	LSC	9	L9	23	1	0
194	LSC	9	L9	23	1	0
195	LSC	9	L9	23	1	0
196	LSC	9	L9	24	1	0
197	LSC	9	L9	24	1	0
198	LSC	9	L9	24	1	0
199	LSC	9	L9	24	1	0
200	LSC	9	L9	24	1	0
201	LSC	9	L9	24	1	0
202	LSC	9	L9	24	1	0
203	LSC	9	L9	24	1	0
204	LSC	9	L9	24	1	0
205	LSC	9	L9	25	1	1
206	LSC	9	L9	25	1	1
207	LSC	9	L9	25	1	1
208	LSC	9	L9	25	1	0
209	LSC	9	L9	25	1	0
210	LSC	9	L9	25	1	0
211	LSC	9	L9	25	1	0
212	LSC	9	L9	25	1	0
213	LSC	9	L9	25	1	0
214	LSC	9	L9	26	1	0
215	LSC	9	L9	26	1	0
216	LSC	9	L9	26	1	0

Fertility data1.sav

	Hatched	Pipped	ED	LD	Dead
181	0	0	0	0	0
182	0	0	0	0	0
183	0	0	0	0	0
184	0	0	0	0	0
185	0	0	0	0	0
186	0	0	0	0	0
187	0	0	0	0	0
188	0	0	0	0	0
189	0	0	0	0	0
190	0	0	0	0	0
191	0	0	0	0	0
192	0	0	0	0	0
193	0	0	0	0	0
194	0	0	0	0	0
195	0	0	0	0	0
196	0	0	0	0	0
197	0	0	0	0	0
198	0	0	0	0	0
199	0	0	0	0	0
200	0	0	0	0	0
201	0	0	0	0	0
202	0	0	0	0	0
203	0	0	0	0	0
204	0	0	0	0	0
205	0	1	0	0	0
206	0	1	0	0	0
207	0	1	0	0	0
208	0	0	0	0	0
209	0	0	0	0	0
210	0	0	0	0	0
211	0	0	0	0	0
212	0	0	0	0	0
213	0	0	0	0	0
214	0	0	0	0	0
215	0	0	0	0	0
216	0	0	0	0	0

Fertility data1.sav

	Group	DMA	Interaction	Hen	Eggs	Fertile
217	LSC	9	L9	26	1	0
218	LSC	9	L9	26	1	0
219	LSC	9	L9	26	1	0
220	LSC	9	L9	26	1	0
221	LSC	9	L9	26	1	0
222	LSC	9	L9	27	1	0
223	LSC	9	L9	27	1	0
224	LSC	9	L9	27	1	0
225	LSC	9	L9	27	1	0
226	LSC	9	L9	27	1	0
227	LSC	9	L9	27	1	0
228	LSC	9	L9	27	1	0
229	LSC	9	L9	27	1	0
230	LSC	9	L9	27	1	0
231	LSC	9	L9	28	1	0
232	LSC	9	L9	28	1	0
233	LSC	9	L9	28	1	0
234	LSC	9	L9	28	1	0
235	LSC	9	L9	28	1	0
236	LSC	9	L9	28	1	0
237	LSC	9	L9	28	1	0
238	LSC	9	L9	28	1	0
239	LSC	9	L9	28	1	0
240	LSC	9	L9	29	1	0
241	LSC	9	L9	29	1	0
242	LSC	9	L9	29	1	0
243	LSC	9	L9	29	1	0
244	LSC	9	L9	29	1	0
245	LSC	9	L9	29	1	0
246	LSC	9	L9	29	1	0
247	LSC	9	L9	29	1	0
248	LSC	9	L9	30	1	1
249	LSC	9	L9	30	1	1
250	LSC	9	L9	30	1	1
251	LSC	9	L9	30	1	0
252	LSC	9	L9	30	1	0

Fertility data1.sav

	Hatched	Pipped	ED	LD	Dead
217	0	0	0	0	0
218	0	0	0	0	0
219	0	0	0	0	0
220	0	0	0	0	0
221	0	0	0	0	0
222	0	0	0	0	0
223	0	0	0	0	0
224	0	0	0	0	0
225	0	0	0	0	0
226	0	0	0	0	0
227	0	0	0	0	0
228	0	0	0	0	0
229	0	0	0	0	0
230	0	0	0	0	0
231	0	0	0	0	0
232	0	0	0	0	0
233	0	0	0	0	0
234	0	0	0	0	0
235	0	0	0	0	0
236	0	0	0	0	0
237	0	0	0	0	0
238	0	0	0	0	0
239	0	0	0	0	0
240	0	0	0	0	0
241	0	0	0	0	0
242	0	0	0	0	0
243	0	0	0	0	0
244	0	0	0	0	0
245	0	0	0	0	0
246	0	0	0	0	0
247	0	0	0	0	0
248	0	1	0	0	0
249	0	1	0	0	0
250	0	1	0	0	0
251	0	0	0	0	0
252	0	0	0	0	0

	Group	DMA	Interaction	Hen	Eggs	Fertile
253	LSC	9	L9	30	1	0
254	LSC	9	L9	30	1	0
255	LSC	9	L9	30	1	0
256	HSC	3	H3	31	1	1
257	HSC	3	H3	31	1	0
258	HSC	3	H3	31	1	0
259	HSC	3	H3	31	1	0
260	HSC	3	H3	31	1	0
261	HSC	3	H3	31	1	0
262	HSC	3	H3	31	1	0
263	HSC	3	H3	31	1	0
264	HSC	3	H3	31	1	0
265	HSC	3	H3	32	1	0
266	HSC	3	H3	32	1	0
267	HSC	3	H3	32	1	0
268	HSC	3	H3	32	1	0
269	HSC	3	H3	32	1	1
270	HSC	3	H3	32	1	0
271	HSC	3	H3	32	1	0
272	HSC	3	H3	32	1	1
273	HSC	3	H3	32	1	1
274	HSC	3	H3	33	1	0
275	HSC	3	H3	33	1	0
276	HSC	3	H3	33	1	1
277	HSC	3	H3	33	1	0
278	HSC	3	H3	33	1	0
279	HSC	3	H3	33	1	0
280	HSC	3	H3	33	1	0
281	HSC	3	H3	33	1	0
282	HSC	3	H3	33	1	0
283	HSC	3	H3	34	1	0
284	HSC	3	H3	34	1	0
285	HSC	3	H3	34	1	0
286	HSC	3	H3	34	1	0
287	HSC	3	H3	34	1	0
288	HSC	3	H3	34	1	0

Fertility data1.sav

	Hatched	Pipped	ED	LD	Dead
253	0	0	0	0	0
254	0	0	0	0	0
255	0	0	0	0	0
256	0	1	0	0	0
257	0	0	0	0	0
258	0	0	0	0	0
259	0	0	0	0	0
260	0	0	0	0	0
261	0	0	0	0	0
262	0	0	0	0	0
263	0	0	0	0	0
264	0	0	0	0	0
265	0	0	0	0	0
266	0	0	0	0	0
267	0	0	0	0	0
268	0	0	0	0	0
269	0	1	0	0	0
270	0	0	0	0	0
271	0	0	0	0	0
272	0	1	0	0	0
273	0	1	0	0	0
274	0	0	0	0	0
275	0	0	0	0	0
276	0	1	0	0	0
277	0	0	0	0	0
278	0	0	0	0	0
279	0	0	0	0	0
280	0	0	0	0	0
281	0	0	0	0	0
282	0	0	0	0	0
283	0	0	0	0	0
284	0	0	0	0	0
285	0	0	0	0	0
286	0	0	0	0	0
287	0	0	0	0	0
288	0	0	0	0	0

Fertility data1.sav

	Group	DMA	Interaction	Hen	Eggs	Fertile
289	HSC	3	H3	34	1	1
290	HSC	3	H3	34	1	0
291	HSC	3	H3	35	1	0
292	HSC	3	H3	35	1	1
293	HSC	3	H3	35	1	1
294	HSC	3	H3	35	1	0
295	HSC	3	H3	35	1	0
296	HSC	3	H3	35	1	0
297	HSC	3	H3	35	1	1
298	HSC	3	H3	35	1	0
299	HSC	3	H3	35	1	0
300	HSC	3	H3	36	1	0
301	HSC	3	H3	36	1	0
302	HSC	3	H3	36	1	0
303	HSC	3	H3	36	1	0
304	HSC	3	H3	36	1	0
305	HSC	3	H3	36	1	0
306	HSC	3	H3	36	1	0
307	HSC	3	H3	36	1	1
308	HSC	3	H3	36	1	1
309	HSC	3	H3	37	1	0
310	HSC	3	H3	37	1	0
311	HSC	3	H3	37	1	0
312	HSC	3	H3	37	1	0
313	HSC	3	H3	37	1	1
314	HSC	3	H3	37	1	1
315	HSC	3	H3	37	1	0
316	HSC	3	H3	37	1	0
317	HSC	3	H3	37	1	0
318	HSC	3	H3	38	1	0
319	HSC	3	H3	38	1	0
320	HSC	3	H3	38	1	1
321	HSC	3	H3	38	1	1
322	HSC	3	H3	38	1	1
323	HSC	3	H3	38	1	0
324	HSC	3	H3	38	1	1

Fertility data1.sav

	Hatched	Pipped	ED	LD	Dead
289	0	1	0	0	0
290	0	0	0	0	0
291	0	0	0	0	0
292	0	1	0	0	0
293	0	1	0	0	0
294	0	0	0	0	0
295	0	0	0	0	0
296	0	0	0	0	0
297	1	0	0	0	0
298	0	0	0	0	0
299	0	0	0	0	0
300	0	0	0	0	0
301	0	0	0	0	0
302	0	0	0	0	0
303	0	0	0	0	0
304	0	0	0	0	0
305	0	0	0	0	0
306	0	0	0	0	0
307	1	0	0	0	0
308	1	0	0	0	0
309	0	0	0	0	0
310	0	0	0	0	0
311	0	0	0	0	0
312	0	0	0	0	0
313	0	1	0	0	0
314	1	0	0	0	0
315	0	0	0	0	0
316	0	0	0	0	0
317	0	0	0	0	0
318	0	0	0	0	0
319	0	0	0	0	0
320	0	0	0	1	1
321	1	0	0	0	0
322	1	0	0	0	0
323	0	0	0	0	0
324	0	1	1	0	1

Fertility data1.sav

	Group	DMA	Interaction	Hen	Eggs	Fertile
325	HSC	3	H3	38	1	1
326	HSC	3	H3	38	1	1
327	HSC	3	H3	39	1	0
328	HSC	3	H3	39	1	0
329	HSC	3	H3	39	1	0
330	HSC	3	H3	39	1	1
331	HSC	3	H3	39	1	1
332	HSC	3	H3	39	1	0
333	HSC	3	H3	39	1	0
334	HSC	3	H3	39	1	0
335	HSC	3	H3	40	1	0
336	HSC	3	H3	40	1	0
337	HSC	3	H3	40	1	1
338	HSC	3	H3	40	1	1
339	HSC	3	H3	40	1	0
340	HSC	3	H3	40	1	0
341	?	3	H3	40	1	0
342	HSC	3	H3	40	1	0
343	HSC	6	H6	41	1	1
344	HSC	6	H6	41	1	0
345	HSC	6	H6	41	1	0
346	HSC	6	H6	41	1	0
347	HSC	6	H6	41	1	0
348	HSC	6	H6	41	1	0
349	HSC	6	H6	41	1	0
350	HSC	6	H6	41	1	0
351	HSC	6	H6	42	1	0
352	HSC	6	H6	42	1	0
353	HSC	6	H6	42	1	1
354	HSC	6	H6	42	1	0
355	HSC	6	H6	42	1	0
356	HSC	6	H6	42	1	0
357	HSC	6	H6	42	1	0
358	HSC	6	H6	42	1	0
359	HSC	6	H6	43	1	0
360	HSC	6	H6	43	1	1

Fertility data1.sav

	Hatched	Pipped	ED	LD	Dead
325	1	0	0	0	0
326	0	0	0	0	0
327	0	0	0	0	0
328	0	0	0	0	0
329	0	0	0	0	0
330	0	1	0	0	0
331	1	0	0	0	0
332	0	0	0	0	0
333	0	0	0	0	0
334	0	0	0	0	0
335	0	0	0	0	0
336	0	0	0	0	0
337	0	1	1	0	1
338	0	0	0	0	0
339	0	0	0	0	0
340	0	0	0	0	0
341	0	0	0	0	0
342	0	0	0	0	0
343	0	1	0	0	0
344	0	0	0	0	0
345	0	0	0	0	0
346	0	0	0	0	0
347	0	0	0	0	0
348	0	0	0	0	0
349	0	0	0	0	0
350	0	0	0	0	0
351	0	0	0	0	0
352	0	0	0	0	0
353	0	1	0	0	0
354	0	0	0	0	0
355	0	0	0	0	0
356	0	0	0	0	0
357	0	0	0	0	0
358	0	0	0	0	0
359	0	0	0	0	0
360	0	1	0	0	0

Fertility data1.sav

	Group	DMA	Interaction	Hen	Eggs	Fertile
361	HSC	6	H6	43	1	1
362	HSC	6	H6	43	1	0
363	HSC	6	H6	43	1	0
364	HSC	6	H6	43	1	0
365	HSC	6	H6	43	1	0
366	HSC	6	H6	43	1	0
367	HSC	6	H6	44	1	0
368	HSC	6	H6	44	1	0
369	HSC	6	H6	44	1	0
370	HSC	6	H6	44	1	0
371	HSC	6	H6	44	1	0
372	HSC	6	H6	44	1	0
373	HSC	6	H6	44	1	0
374	HSC	6	H6	44	1	1
375	HSC	6	H6	45	1	1
376	HSC	6	H6	45	1	0
377	HSC	6	H6	45	1	0
378	HSC	6	H6	45	1	0
379	HSC	6	H6	45	1	0
380	HSC	6	H6	45	1	0
381	HSC	6	H6	45	1	0
382	HSC	6	H6	45	1	0
383	HSC	6	H6	46	1	1
384	HSC	6	H6	46	1	0
385	HSC	6	H6	46	1	0
386	HSC	6	H6	46	1	0
387	HSC	6	H6	46	1	1
388	HSC	6	H6	46	1	1
389	HSC	6	H6	46	1	0
390	HSC	6	H6	46	1	0
391	HSC	6	H6	47	1	0
392	HSC	6	H6	47	1	0
393	HSC	6	H6	47	1	0
394	HSC	6	H6	47	1	0
395	HSC	6	H6	47	1	0
396	HSC	6	H6	47	1	0

...	Hatched	Pipped	ED	LD	Dead
361	1	0	0	0	0
362	0	0	0	0	0
363	0	0	0	0	0
364	0	0	0	0	0
365	0	0	0	0	0
366	0	0	0	0	0
367	0	0	0	0	0
368	0	0	0	0	0
369	0	0	0	0	0
370	0	0	0	0	0
371	0	0	0	0	0
372	0	0	0	0	0
373	0	0	0	0	0
374	0	1	0	0	0
375	1	0	0	0	0
376	0	0	0	0	0
377	0	0	0	0	0
378	0	0	0	0	0
379	0	0	0	0	0
380	0	0	0	0	0
381	0	0	0	0	0
382	0	0	0	0	0
383	0	1	0	0	0
384	0	0	0	0	0
385	0	0	0	0	0
386	0	0	0	0	0
387	0	1	0	0	0
388	1	0	0	0	0
389	0	0	0	0	0
390	0	0	0	0	0
391	0	0	0	0	0
392	0	0	0	0	0
393	0	0	0	0	0
394	0	0	0	0	0
395	0	0	0	0	0
396	0	0	0	0	0

Fertility data1.sav

	Group	DMA	Interaction	Hen	Eggs	Fertile
397	HSC	6	H6	47	1	0
398	HSC	6	H6	47	1	0
399	HSC	6	H6	48	1	0
400	HSC	6	H6	48	1	0
401	HSC	6	H6	48	1	1
402	HSC	6	H6	48	1	1
403	HSC	6	H6	48	1	1
404	HSC	6	H6	48	1	0
405	HSC	6	H6	48	1	0
406	HSC	6	H6	48	1	0
407	HSC	6	H6	48	1	0
408	HSC	6	H6	49	1	1
409	HSC	6	H6	49	1	0
410	HSC	6	H6	49	1	0
411	HSC	6	H6	49	1	0
412	HSC	6	H6	49	1	0
413	HSC	6	H6	49	1	0
414	HSC	6	H6	49	1	0
415	HSC	6	H6	49	1	1
416	HSC	6	H6	49	1	0
417	HSC	6	H6	50	1	0
418	HSC	6	H6	50	1	0
419	HSC	6	H6	50	1	0
420	HSC	6	H6	50	1	0
421	HSC	6	H6	50	1	0
422	HSC	6	H6	50	1	1
423	HSC	6	H6	50	1	0
424	HSC	6	H6	50	1	0
425	HSC	6	H6	50	1	0
426	HSC	9	H9	51	1	1
427	HSC	9	H9	51	1	1
428	HSC	9	H9	51	1	1
429	HSC	9	H9	51	1	0
430	HSC	9	H9	51	1	0
431	HSC	9	H9	51	1	1
432	HSC	9	H9	51	1	0

	Hatched	Pipped	ED	LD	Dead
397	0	0	0	0	0
398	0	0	0	0	0
399	0	0	0	0	0
400	0	0	0	0	0
401	0	0	1	0	1
402	0	1	0	0	0
403	1	0	0	0	0
404	0	0	0	0	0
405	0	0	0	0	0
406	0	0	0	0	0
407	0	0	0	0	0
408	1	0	0	0	0
409	0	0	0	0	0
410	0	0	0	0	0
411	0	0	0	0	0
412	0	0	0	0	0
413	0	0	0	0	0
414	0	0	0	0	0
415	0	0	1	0	1
416	0	0	0	0	0
417	0	0	0	0	0
418	0	0	0	0	0
419	0	0	0	0	0
420	0	0	0	0	0
421	0	0	0	0	0
422	0	1	0	0	0
423	0	0	0	0	0
424	0	0	0	0	0
425	0	0	0	0	0
426	0	0	1	0	1
427	0	1	0	0	0
428	1	0	0	0	0
429	0	0	0	0	0
430	0	0	0	0	0
431	0	1	0	0	0
432	0	0	0	0	0

Fertility data1.sav

	Group	DMA	Interaction	Hen	Eggs	Fertile
433	HSC	9	H9	51	1	0
434	HSC	9	H9	51	1	0
435	HSC	9	H9	52	1	0
436	HSC	9	H9	52	1	0
437	HSC	9	H9	52	1	1
438	HSC	9	H9	52	1	0
439	HSC	9	H9	52	1	0
440	HSC	9	H9	52	1	0
441	HSC	9	H9	52	1	0
442	HSC	9	H9	52	1	0
443	HSC	9	H9	53	1	1
444	HSC	9	H9	53	1	1
445	HSC	9	H9	53	1	1
446	HSC	9	H9	53	1	0
447	HSC	9	H9	53	1	1
448	HSC	9	H9	53	1	0
449	HSC	9	H9	53	1	0
450	HSC	9	H9	53	1	0
451	HSC	9	H9	53	1	0
452	HSC	9	H9	54	1	1
453	HSC	9	H9	54	1	1
454	HSC	9	H9	54	1	0
455	HSC	9	H9	54	1	0
456	HSC	9	H9	54	1	0
457	HSC	9	H9	54	1	1
458	HSC	9	H9	54	1	0
459	HSC	9	H9	54	1	0
460	HSC	9	H9	54	1	0
461	HSC	9	H9	55	1	0
462	HSC	9	H9	55	1	0
463	HSC	9	H9	55	1	1
464	HSC	9	H9	55	1	1
465	HSC	9	H9	55	1	0
466	HSC	9	H9	55	1	0
467	HSC	9	H9	55	1	0
468	HSC	9	H9	55	1	0

Fertility data1.sav

	Hatched	Pipped	ED	LD	Dead
433	0	0	0	0	0
434	0	0	0	0	0
435	0	0	0	0	0
436	0	0	0	0	0
437	0	0	1	0	1
438	0	0	0	0	0
439	0	0	0	0	0
440	0	0	0	0	0
441	0	0	0	0	0
442	0	0	0	0	0
443	0	0	1	0	1
444	0	1	0	0	0
445	0	1	0	0	0
446	0	0	0	0	0
447	0	1	0	0	0
448	0	0	0	0	0
449	0	0	0	0	0
450	0	0	0	0	0
451	0	0	0	0	0
452	0	0	1	0	1
453	0	1	0	0	0
454	0	0	0	0	0
455	0	0	0	0	0
456	0	0	0	0	0
457	1	0	0	0	0
458	0	0	0	0	0
459	0	0	0	0	0
460	0	0	0	0	0
461	0	0	0	0	0
462	0	0	0	0	0
463	0	0	0	1	1
464	0	1	0	0	0
465	0	0	0	0	0
466	0	0	0	0	0
467	0	0	0	0	0
468	0	0	0	0	0

Fertility data1.sav

	Group	DMA	Interaction	Hen	Eggs	Fertile
469	HSC	9	H9	56	1	0
470	HSC	9	H9	56	1	0
471	HSC	9	H9	56	1	0
472	HSC	9	H9	56	1	0
473	HSC	9	H9	56	1	0
474	HSC	9	H9	56	1	0
475	HSC	9	H9	56	1	0
476	HSC	9	H9	56	1	0
477	HSC	9	H9	57	1	1
478	HSC	9	H9	57	1	1
479	HSC	9	H9	57	1	0
480	HSC	9	H9	57	1	0
481	HSC	9	H9	57	1	0
482	HSC	9	H9	57	1	0
483	HSC	9	H9	57	1	0
484	HSC	9	H9	57	1	0
485	HSC	9	H9	57	1	0
486	HSC	9	H9	58	1	0
487	HSC	9	H9	58	1	0
488	HSC	9	H9	58	1	1
489	HSC	9	H9	58	1	0
490	HSC	9	H9	58	1	0
491	HSC	9	H9	58	1	0
492	HSC	9	H9	58	1	0
493	HSC	9	H9	58	1	0
494	HSC	9	H9	59	1	0
495	HSC	9	H9	59	1	0
496	HSC	9	H9	59	1	0
497	HSC	9	H9	59	1	0
498	HSC	9	H9	59	1	0
499	HSC	9	H9	59	1	0
500	HSC	9	H9	59	1	0
501	HSC	9	H9	59	1	0
502	HSC	9	H9	59	1	0
503	HSC	9	H9	60	1	0
504	HSC	9	H9	60	1	1

Fertility data1.sav

	Hatched	Pipped	ED	LD	Dead
469	0	0	0	0	0
470	0	0	0	0	0
471	0	0	0	0	0
472	0	0	0	0	0
473	0	0	0	0	0
474	0	0	0	0	0
475	0	0	0	0	0
476	0	0	0	0	0
477	0	1	0	0	0
478	1	0	0	0	0
479	0	0	0	0	0
480	0	0	0	0	0
481	0	0	0	0	0
482	0	0	0	0	0
483	0	0	0	0	0
484	0	0	0	0	0
485	0	0	0	0	0
486	0	0	0	0	0
487	0	0	0	0	0
488	1	0	0	0	0
489	0	0	0	0	0
490	0	0	0	0	0
491	0	0	0	0	0
492	0	0	0	0	0
493	0	0	0	0	0
494	0	0	0	0	0
495	0	0	0	0	0
496	0	0	0	0	0
497	0	0	0	0	0
498	0	0	0	0	0
499	0	0	0	0	0
500	0	0	0	0	0
501	0	0	0	0	0
502	0	0	0	0	0
503	0	0	0	0	0
504	0	0	1	0	1

Fertility data1.sav

	Group	DMA	Interaction	Hen	Eggs	Fertile
505	HSC	9	H9	60	1	1
506	HSC	9	H9	60	1	1
507	HSC	9	H9	60	1	0
508	HSC	9	H9	60	1	0
509	HSC	9	H9	60	1	0
510	HSC	9	H9	60	1	0
511	HSC	9	H9	60	1	0

Fertility data1.sav

	Hatched	Pipped	ED	LD	Dead
505	0	1	0	0	0
506	1	0	0	0	0
507	0	0	0	0	0
508	0	0	0	0	0
509	0	0	0	0	0
510	0	0	0	0	0
511	0	0	0	0	0
