# Supplementary material for: Effect of semen dilution rate and dimethyl acetamide levels on post-thaw motility and fertility parameters of rooster sperm
Source: PLoS One. 2025 Oct 31;20(10):e0335748. doi: 10.1371/journal.pone.0335748 (PMC12578245; doi:10.1371/journal.pone.0335748)
Supplement: Attachment — Submitted filename: PONE-D-25-33278.pdf [file pone.0335748.s003.pdf]

PLOS ONE

Effect of pre-freezing semen dilution rate and cryo-protection with dimethyl acetamide on post-thaw motility and fertility traits of rooster sperm

--Manuscript Draft--

**Manuscript Number:**	PONE-D-25-33278
**Article Type:**	Research Article
**Full Title:**	Effect of pre-freezing semen dilution rate and cryo-protection with dimethyl acetamide on post-thaw motility and fertility traits of rooster sperm
**Short Title:**	Pre-freezing semen dilution rate and cryo-protection with dimethyl acetamide in rooster sperm
**Corresponding Author:**	Gamal M. K. Mehaisen, Ph.D.<br>Cairo University Faculty of Agriculture<br>Giza, EGYPT
**Keywords:**	chicken roosters; semen dilution; dimethyl acetamide; cryopreservation; sperm motility; fertilizing ability
**Abstract:**	This study evaluated the impact of pre-freezing semen dilution rate and dimethyl acetamide (DMA) concentration on the post-thaw motility and fertility of cryopreserved rooster sperm. Rooster ejaculates were diluted with a standard EK extender to achieve low (LSC; 1×109 sperm/mL) and high (HSC; 2×109 sperm/mL) sperm concentrations. Each dilution group was further treated with three DMA concentrations (3%, 6%, or 9%) before cryopreservation. Post-thaw sperm motility traits were obtained by computer-assisted sperm analysis (CASA), and fertility features were evaluated through artificial insemination in hens. The current results showed that HSC significantly improved total motility (TM), curvilinear velocity (VCL), and amplitude of lateral head displacement (ALH), but reduced linearity (LIN) and straightness (STR) compared to LSC. DMA concentration had a quadratic effect on motility, with 6% yielding the highest progressive motility (PM) and beat cross frequency (BCF). Fertility outcomes revealed that HSC resulted in higher fertilization rates, while LSC improved hatchability of fertile eggs. Combining 6% DMA with HSC achieved the best compromise between motility and fertilization, whereas LSC with 6% DMA showed optimal hatchability. These findings underscore the critical interplay between dilution rate and cryoprotectant concentration for optimizing cryopreservation protocols in poultry.
**Order of Authors:**	Mohamed Shawky
	Ahmed F. Fawy
	Ahmed M. Elomda
	Mohamed A. Elmenawey
	Abd El-Rahman M. Atta
	Ahmed O. Abbas
	Gamal M. K. Mehaisen, Ph.D.
**Additional Information:**	
**Question**	**Response**
**Financial Disclosure**<br><br>Enter a financial disclosure statement that describes the sources of funding for the work included in this submission. Review the submission guidelines for detailed requirements. View published research articles from *PLOS ONE* for specific	Yes

Powered by Editorial Manager® and ProduXion Manager® from Aries Systems Corporation

examples.<br><br>This statement is required for submission and **will appear in the published article** if the submission is accepted. Please make sure it is accurate.<br><br>**Funded studies**<br>Enter a statement with the following details:<br>• Initials of the authors who received each award<br>• Grant numbers awarded to each author<br>• The full name of each funder<br>• URL of each funder website<br>• Did the sponsors or funders play any role in the study design, data collection and analysis, decision to publish, or preparation of the manuscript?<br><br>Did you receive funding for this work?	
Please add funding details.<br>as follow-up to "**Financial Disclosure**<br><br>Enter a financial disclosure statement that describes the sources of funding for the work included in this submission. Review the submission guidelines for detailed requirements. View published research articles from *PLOS ONE* for specific examples.<br><br>This statement is required for submission and **will appear in the published article** if the submission is accepted. Please make sure it is accurate.<br><br>**Funded studies**<br>Enter a statement with the following details:<br>• Initials of the authors who received each award<br>• Grant numbers awarded to each author<br>• The full name of each funder<br>• URL of each funder website<br>• Did the sponsors or funders play any role in the study design, data collection and analysis, decision to publish, or preparation of the manuscript?	This research was financially supported by the funds from the Academy of Scientific Research and Technology, Egypt (ASRT 5105) and the Vice Presidency for Graduate Studies and Scientific Research at King Faisal University, Saudi Arabia (Project KFU252322).

Powered by Editorial Manager® and ProduXion Manager® from Aries Systems Corporation

Did you receive funding for this work?"	
Please select the country of your main research funder (please select carefully as in some cases this is used in fee calculation).<br>as follow-up to "**Financial Disclosure**<br><br>Enter a financial disclosure statement that describes the sources of funding for the work included in this submission. Review the submission guidelines for detailed requirements. View published research articles from *PLOS ONE* for specific examples.<br><br>This statement is required for submission and **will appear in the published article** if the submission is accepted. Please make sure it is accurate.<br><br>**Funded studies**<br>Enter a statement with the following details:<br>• Initials of the authors who received each award<br>• Grant numbers awarded to each author<br>• The full name of each funder<br>• URL of each funder website<br>• Did the sponsors or funders play any role in the study design, data collection and analysis, decision to publish, or preparation of the manuscript?<br><br>Did you receive funding for this work?"	EGYPT - EG
**Competing Interests**<br><br>Use the instructions below to enter a competing interest statement for this submission. On behalf of all authors, disclose any competing interests that could be perceived to bias this work—acknowledging all financial support and any other relevant financial or non-financial competing interests.	The authors have declared that no competing interests exist.

This statement is required for submission and **will appear in the published article** if the submission is accepted. Please make sure it is accurate and that any funding sources listed in your Funding Information later in the submission form are also declared in your Financial Disclosure statement.<br><br>View published research articles from *PLOS ONE* for specific examples.<br><br>**NO authors have competing interests**<br><br>Enter: *The authors have declared that no competing interests exist.*<br><br>**Authors with competing interests**<br><br>Enter competing interest details beginning with this statement:<br><br>*I have read the journal's policy and the authors of this manuscript have the following competing interests: [insert competing interests here]*<br><br>* typeset	
**Ethics Statement**<br><br>Enter an ethics statement for this submission. This statement is required if the study involved:<br><br>• Human participants<br>• Human specimens or tissue<br>• Vertebrate animals or cephalopods<br>• Vertebrate embryos or tissues<br>• Field research<br><br>Write "N/A" if the submission does not require an ethics statement.<br><br>General guidance is provided below. Consult the submission guidelines for detailed instructions. **Make sure that all information entered here is included in the Methods section of the manuscript.**	All experimental protocols were authorized by the Cairo University-Institutional Animal Care and Use Committee (Approval No. CU-II-F-12-20).

**Format for specific study types**<br><br>**Human Subject Research (involving human participants and/or tissue)**<br>• Give the name of the institutional review board or ethics committee that approved the study<br>• Include the approval number and/or a statement indicating approval of this research<br>• Indicate the form of consent obtained (written/oral) or the reason that consent was not obtained (e.g. the data were analyzed anonymously)<br><br>**Animal Research (involving vertebrate animals, embryos or tissues)**<br>• Provide the name of the Institutional Animal Care and Use Committee (IACUC) or other relevant ethics board that reviewed the study protocol, and indicate whether they approved this research or granted a formal waiver of ethical approval<br>• Include an approval number if one was obtained<br>• If the study involved *non-human primates*, add *additional details* about animal welfare and steps taken to ameliorate suffering<br>• If anesthesia, euthanasia, or any kind of animal sacrifice is part of the study, include briefly which substances and/or methods were applied<br><br>**Field Research**<br><br>Include the following details if this study involves the collection of plant, animal, or other materials from a natural setting:<br>• Field permit number<br>• Name of the institution or relevant body that granted permission	
**Data Availability**<br><br>Authors are required to make all data underlying the findings described fully available, without restriction, and from the time of publication. PLOS allows rare exceptions to address legal and ethical concerns. See the PLOS Data Policy and FAQ for detailed information.	Yes - all data are fully available without restriction

A Data Availability Statement describing where the data can be found is required at submission. Your answers to this question constitute the Data Availability Statement and **will be published in the article**, if accepted.<br><br>**Important:** Stating 'data available on request from the author' is not sufficient. If your data are only available upon request, select 'No' for the first question and explain your exceptional situation in the text box.<br><br>Do the authors confirm that all data underlying the findings described in their manuscript are fully available without restriction?	
**Describe where the data may be found in full sentences. If you are copying our sample text, replace any instances of XXX with the appropriate details.**<br><br>• If the data are **held or will be held in a public repository**, include URLs, accession numbers or DOIs. If this information will only be available after acceptance, indicate this by ticking the box below. For example: *All XXX files are available from the XXX database (accession number(s) XXX, XXX.*).<br>• If the data are all contained **within the manuscript and/or Supporting Information files**, enter the following: *All relevant data are within the manuscript and its Supporting Information files.*<br>• If neither of these applies but you are able to provide **details of access elsewhere**, with or without limitations, please do so. For example:<br><br>*Data cannot be shared publicly because of [XXX]. Data are available from the XXX Institutional Data Access / Ethics Committee (contact via XXX) for researchers who meet the criteria for access to confidential data.*<br><br>*The data underlying the results presented in the study are available*	All relevant data are within the manuscript and its Supporting Information files.

from (include the name of the third party and contact information or URL).<br>• This text is appropriate if the data are owned by a third party and authors do not have permission to share the data.<br><br>* typeset	
Additional data availability information:	

Powered by Editorial Manager® and ProduXion Manager® from Aries Systems Corporation

Manuscript

Click here to access/download;Manuscript;Final-Fawy-MS-Plos 2025.docx

Effect of pre-freezing semen dilution rate and cryo-protection with dimethyl acetamide on post-thaw motility and fertility traits of rooster sperm

Mohamed Shawky[1], Ahmed F. Fawy[2], Ahmed M. Elomda[3], Mohamed A. Elmenawey[2], Abd El-Rahman M. Atta[2], Ahmed O. Abbas[4], Gamal M. K. Mehaisen[2,*]

[1] Avian Research Center, King Faisal University, P.O. Box 400, Al-Hofuf, Al-Ahsa, 31982, Saudi Arabia

[2] Department of Animal Production, Faculty of Agriculture, Cairo University, Giza, Egypt

[3] Department of Animal Biotechnology, Animal Production Research Institute, Agriculture Research Center, Dokki, Giza, Egypt

[4] Department of Animal and Fish Production, College of Agricultural and Food Sciences, King Faisal University, Al-Ahsa, Saudi Arabia

[*] Corresponding authors: gamoka@cu.edu.eg (GMKM)

Abstract

This study evaluated the impact of pre-freezing semen dilution rate and dimethyl acetamide (DMA) concentration on the post-thaw motility and fertility of cryopreserved rooster sperm. Rooster ejaculates were diluted with a standard EK extender to achieve low (LSC; 1×10^9 sperm/mL) and high (HSC; 2×10^9 sperm/mL) sperm concentrations. Each dilution group was further treated with three DMA concentrations (3%, 6%, or 9%) before cryopreservation. Post-thaw sperm motility traits were obtained by computer-assisted sperm analysis (CASA), and fertility features were evaluated through artificial insemination in hens. The current results showed that HSC significantly improved total motility (TM), curvilinear velocity (VCL), and amplitude of lateral head displacement (ALH), but reduced linearity (LIN) and straightness (STR) compared to LSC. DMA concentration had a quadratic effect on motility, with 6% yielding the highest progressive motility (PM) and beat cross frequency (BCF). Fertility outcomes revealed that HSC resulted in higher fertilization rates, while LSC improved hatchability of fertile eggs. Combining 6% DMA with HSC achieved the best compromise between motility and fertilization, whereas LSC with 6% DMA showed optimal hatchability. These findings underscore the critical interplay between dilution rate and cryoprotectant concentration for optimizing cryopreservation protocols in poultry.

Key words: chicken roosters, semen dilution, dimethyl acetamide, cryopreservation, sperm motility, fertilizing ability

Introduction

Sperm cryopreservation remains the most applicable approach for facilitating artificial insemination, disease control, breeding, and selection programs in poultry species [1]. However, the process of sperm freezing is intricate. It relies on several essential steps, either

those affecting the quality of semen samples, such as male performance, ejaculate collection, manipulation time, extender composition, and dilution rate, or those correlated with the freezing technique per se, including cryoprotectant (CPA) concentration, CPA equilibration, packaging type, freezing rate, CPA removing (if glycerol is utilized), and thawing procedure [2]. The previous results have shown that frozen/thawed semen in the poultry industry remains restricted and not economically feasible because avian spermatozoa exhibit high levels of sensitivity, damage, and infertility rates after undergoing cryopreservation procedures [3].

Dimethyl acetamide (DMA), consisting of one amide group connected to two methyl groups [CH_3-C(=O)-N(CH_3)$_2$], is commonly used as an intracellular cryoprotectant for semen freezing in poultry species [4, 5]. It is suggested that DMA can penetrate cell membranes rapidly and participate in the natural biological processes of the cells [6]. DMA freezing technology is based on its capacity to substitute the intracellular water of sperm cells, thereby mitigating the harms caused by ice formation [7]. In contrast, the initial interaction of sperm with DMA during the freezing/thawing process may influence the membrane fluidity and acrosome reaction, probably exerting adverse effects on post-thaw sperm viability [8, 9]. Earlier studies implemented DMA with semen extenders at wide levels ranging from 3-26% to boost the effectiveness of chicken sperm cryopreservation [10-19]. However, it was found that an appropriate sperm motility across various poultry species was obtained when applying semen freezing with a low concentration of DMA not exceeding 3-6% [12, 13, 17, 18]. In contrast, the average fertility levels obtained from cryopreserved chicken semen utilizing DMA remain low and exhibit considerable variability, ranging from 10% to 77% [14, 15, 17, 20].

The dilution of semen is a valuable approach for evaluating and/or increasing the fertilizing capability and hatchability of individual broiler breeder males [21-23]. It is well known that semen dilution may affect the viscosity, in turn, affecting sperm motility [21]. Standardizing semen dilution at the initiation stage of cryopreservation protocols is essential

because of the individual variation in ejaculated sperm concentration from the birds [24]. Studies show that the sperm count in fresh semen impacts the quality of turkey semen pellets after cryopreservation [25].

According to Zaniboni, Cassinelli (13), sperm concentration in rooster semen affected sperm motility and viability during semen processing. They reported that high sperm dilution to 1×109 sperm/mL had a beneficial impact on sperm motility. Due to the variation in the amount of sperm cells per milliliter of seminal plasma and extender, Iaffaldano, Romagnoli (26) discovered that a dilution rate 1:4 was more successful than 1:3 in turkey semen. Moreover, Iaffaldano, Manchisi (27) observed that pre-freezing sperm concentration influenced the cryosurvival and quality of post-thaw sperm cells in turkey. They also ascribed this impact to variations in the levels of DMA in each sperm cell or in the amounts of seminal plasma in the sperm sample's overall volume. Furthermore, seminal plasma appears to have a detrimental impact on sperm cells during both liquid storage [28, 29] and cryopreservation [26]. It has been suggested that extracting seminal plasma from turkey spermatozoa prior to semen freezing might increase reproduction rates [1].

To our knowledge, no studies have conclusively demonstrated an optimal dilution rate for chicken semen prior to the freezing procedure with various levels of dimethyl acetamide as a cryoprotectant for chicken sperm cryopreservation. Consequently, this study aimed to investigate the effect of pre-freezing semen dilution rates on post-thaw sperm motility and fertilizing ability of rooster semen cryopreserved with different levels of dimethyl acetamide.

Materials and methods

Ethical statement

This experiment was conducted at the Agricultural Experiments Station (Cairo University). All experimental protocols were authorized by the Cairo University-Institutional Animal Care and Use Committee (CU-II-F-12-20).

Management of birds

A total of sixteen roosters (55-wk-old) and eighty hens (40-wk-old) of Cairo-B2 chicken strain [18] were employed for the current study. The birds were placed in individual battery cages (50×50×60 cm) and fed on a standard commercial breeder ration containing 14% crude protein, 2750 Kcal metabolizable energy, 4.15% crude fiber, and 3.3% crude fat (Feedmix-Egypt Co., Obour city, Kaliobeya, Egypt).

Experimental design

Semen was ejaculated from the roosters twice a week by a lateral pressing of the cloaca after a few seconds of dorso-abdominal massage, as Bakst and Dymond (30) outlined. Semen ejaculates of the first two weeks were neglected, and then the semen ejaculates for 4 consecutive weeks were received in sterilized glass tubes. The individual ejaculates were selected subjectively based on the high gross motility and concentration to be pooled. An objective evaluation based on sperm concentration and progressive motility was then performed on each pool, as mentioned later. The semen pools with at least 4×10^9 sperm/mL and 60% progressive motility were divided into two groups according to the dilution rate at low (LSC) and high (HSC) sperm concentration. The LSC and HSC groups were diluted with a standard EK semen extender until reaching 1×10^9 and 2×10^9 sperm/mL, respectively. The EK extender for semen consisted of 1.4 g of sodium glutamate, 0.98 g of disodium hydrogen phosphate, 0.7 g of glucose, 0.7 g of inositol, 0.21 g of sodium di-hydrogen phosphate, 0.2 g of D-fructose, 0.14 g of potassium citrate, 0.1 g of polyvinylpyrrolidone, and 0.02 g of

protamine sulfate, all dissolved in 100 ml of bi-distilled water (385 mOsmol/kg and 7.8 pH). [31]. The semen pools in both groups were cooled for one hour at 5 °C and then, each divided into three subgroups and equilibrated for a further 10 min at 5 °C with a precooled DMA solution (Qualikems Fine Chem Pvt. Ltd., Vadodara, India) at a final concentration of 3%, 6%, or 9%, respectively. After that, the semen samples were uploaded into mini straws of 250 µL (IMV Technologies, L'Aigle, France). The semen was frozen by maintaining the straws horizontally at 5 cm over the liquid nitrogen (LN_2) surface for 10 min, then immersed in the LN_2. The frozen semen was thawed after 3 months of storage in the LN_2 tank. For semen thawing, the straws were submerged in a water bath at 38 °C for 10 seconds.

Detection of sperm concentration and dilution rate

Five µL of the semen pool was diluted with 995 µL of phosphate-buffered saline (PBS) containing 10% eosin (Bio-Diagnostic, Inc., Giza, Egypt). An aliquot of the dilution was pipetted onto a hemocytometer slide and scanned under a microscope at a magnification of 400×. The number of sperm in five large squares (ns) was counted to express the sperm concentration as ns×10^7 cells per mL. Immediately after determining the actual sperm concentration, the semen pool was evenly divided into two clean tubes, and a calculated volume of EK extender was added to each tube to achieve the working dilution rates for the LSC and HSC groups, respectively.

Motility traits

After dilution with pre-warmed EK extender to a final ratio of 1:50 (v/v), an aliquot of the post-thaw semen sample from each treatment subgroup was selected for computer-assisted sperm analysis (CASA; SpermVision™ software, Minitube GmbH, Tiefenbach, Germany) to assess sperm motility. A minimum of five randomly selected microscopic fields (300–500 sperm per field) were captured at 60 frames per second using a negative high-contrast

microscope (Olympus-BX, Tokyo, Japan) at a total magnification of 200x. The CASA system was configured to analyze these fields. Total motile sperm (TM) percentage, progressively motile sperm (PM) percentage, average path velocity (VAP, µm/sec), curvilinear line velocity (VCL, µm/sec), straight line velocity (VSL, µm/sec), straightness (STR = VSL/VAP %), linearity (LIN = VSL/VCL %), wobble (WOB = VAP/VCL %), amplitude of lateral head displacement (ALH, µm), and beat cross frequency (BCF, Hz) were among the data evaluated by the CASA. The CASA criteria required VSL > 5 µm/sec for motile rooster sperm, and VAP > 20 µm/sec and STR > 80% for progressive motile sperm.

Fertility traits

Ten hens were allocated for artificial insemination with the thawed semen from each treatment subgroup. Each hen received 3 intravaginal inseminations at 2-day intervals with approximately $300×10^6$ sperm using a micropipett[illegible]gs were collected for 8 consecutive days starting from the 2nd day of initiating the insemination. The collected eggs were arranged into setter trays manufactured locally by the Poultry Technical Office (PTO, Alexandria, Egypt) and incubated for 17 days (99.6 °F temperature and 58% humidity). Then, the eggs were transferred to a hatcher (PTO) and incubated for 3 days (98.5 °F and 92 °F in dry and wet bulb readings). Upon completion of the incubation period, the quantity of hatched eggs was noted. Unhatched eggs were cracked and assorted into infertile eggs, early-dead, late-dead, and pipped embryos.

Statistical analysis

Eight semen pools were considered to explore the data on sperm motility and fertility in the present study. Data were randomly arranged under the two dilution rates (LC and HC) as the primary treatment groups, each containing the same DMA concentration subgroups (3, 6, and 9%). All statistical analysis procedures were performed using IBM SPSS Statistics 22.

The effect of dilution rates, DMA concentrations, and their interactions on the sperm motility parameters was analyzed using multivariate General Linear Model (GLM) procedures. The mean values were compared using Tukey's post-hoc test, considering the significance level at $p < 0.05$. A polynomial contrast assay was included in this analysis to examine the linear and quadratic effects of increasing DMA concentration. The fertility traits in all treatment groups were analyzed using Chi-Square (X^2) tests, considering that values differ significantly at $p < 0.05$.

Results

Sperm motility

As shown in Table 1, there is a significant increase in the post-thaw sperm TM, VCL, ALH, and BCF, and a substantial decrease in the LIN, STR, and WOB for the dilution rate at LSC versus HSC (p<0.05). There is a considerable increase in the post-thaw sperm TM, VCL, ALH, and BCF, along with a substantial decrease in LIN, STR, and WOB for the dilution rate at LSC compared to HSC (p<0.05). Regardless of dilution rates, DMA concentration significantly (p<0.05) influenced the post-thaw sperm motility parameters, except for ALH. A notable quadratic trend was observed for DMA concentration regarding sperm TM, PM, VSL, and BCF, with the highest values at 6% DMA compared to 3% or 9% DMA. Conversely, increasing DMA concentration from 3% to 9% resulted in a linear decrease (p<0.05) in VCL and a linear increase in LIN, STR, and WOB. Additionally, a significant (p<0.05) interaction effect was noted in sperm VCL, LIN, and STR. The dilution rate at LSC significantly reduced VCL compared to the HSC dilution group, particularly when using 9% DMA. Conversely, LIN and STR were considerably higher in the LSC group, especially when using 6% DMA, than in the other groups.

Tab[illegible] 1. Effect of semen dilution rate on post-thaw motility parameters [illegible] rooster sperm frozen with various concentrations of dimethyl acetamide.

Treatment Groups	n	TM, %	PM, %	VCL, µm/s	VSL, µm/s	LIN, %	STR, %	WOB, %	ALH, µm	BCF, Hz
**Dilution rate**										
**LSC**	72	44.4 [b]	27.5	92.9 [b]	35.4	37.8 [a]	65.9 [a]	57.1 [a]	4.4 [b]	25.3 [b]
**HSC**	72	54.5 [a]	27.4	122.5 [a]	36.0	29.7 [b]	57.2 [b]	51.4 [b]	5.5 [a]	27.2 [a]
**±SEM**		2.78	1.96	4.99	1.51	1.20	1.40	1.00	0.14	0.79
**DMA concentration**										
**3%**	36	46.5 [b]	24.3 [b]	113.2 [a]	34.7 [b]	31.7 [b]	59.8 [b]	52.3 [b]	5.0	26.1 [b]
**6%**	36	54.9 [a]	33.5 [a]	112.9 [a]	38.0 [a]	34.0 [a]	61.4 [ab]	54.9 [a]	5.0	27.5 [a]
**9%**	36	46.9 [b]	24.5 [b]	96.9 [b]	34.5 [b]	35.5 [a]	63.3 [a]	55.5 [a]	4.8	25.1 [b]
**±SEM**		2.41	1.70	4.33	1.31	1.10	1.20	0.90	0.12	0.69
**Interaction**										
**LSC × 3%**	18	40.4	23.5	93.4 [bc]	34.5	36.6 [ab]	65.5 [a]	55.6	4.5	24.6
**LSC × 6%**	18	49.5	33.8	98.1 [bc]	38.4	39.0 [a]	66.9 [a]	58.3	4.4	26.6
**LSC × 9%**	18	43.3	25.3	87.2 [c]	33.3	37.8 [a]	65.3 [a]	57.4	4.4	24.7
**HSC × 3%**	18	52.6	25.2	133.1 [a]	35.0	26.8 [c]	54.2 [c]	49.0	5.6	27.7
**HSC × 6%**	18	60.3	33.2	127.8 [a]	37.5	29.1 [c]	56.0 [c]	51.5	5.5	28.3
**HSC × 9%**	18	50.6	23.8	106.7 [b]	35.7	33.2 [b]	61.3 [b]	53.7	5.3	25.5
**±SEM**		3.88	2.73	6.97	2.11	1.70	2.00	1.50	0.20	1.11
**p-value**										

**Dilution rate**		<0.001	0.885	<0.001	0.448	<0.001	<0.001	<0.001	<0.001	<0.001
**DMA concentration**		<0.001	<0.001	<0.001	0.001	<0.001	0.002	<0.001	0.266	<0.001
**Interaction**		0.440	0.466	0.017	0.296	0.002	<0.001	0.071	0.578	0.118
**DMA linear effect**		0.834	0.871	<0.001	0.833	<0.001	<0.001	<0.001	0.112	0.067
**DMA quadratic effect**		<0.001	<0.001	0.009	<0.001	0.552	0.867	0.119	0.714	<0.001

Abbreviations: LSC, dilution rate at low sperm concentration; HSC, dilution rate at high sperm concentration; DMA, dimethyl acetamide; TM, total motility; PM: progressive motility; VCL: velocity curved line (µm/s); VSL: velocity straight line (µm/s); STR: LIN: linearity (VSL/VCL %); straightness (VSL/VAP %); WOB: wobble (VAP/VCL %); ALH: amplitude of lateral head displacement (µm); BCF: beat cross frequency (Hz). Data are presented as means for dilution rate (LSC versus HSC), DMA concentration (3, 6, and 9%), and their interaction ± standard error of means (SEM). Means with different superscripts, within a parameter and treatment group, are significantly different ($P<0.05$).

Sperm fertility

The fertility of the HSC group was significantly ($p<0.05$) higher than that of the LSC group (Table 2). On the other hand, the hatchability of fertile eggs was greater in the LSC group than in the HSC group ($p<0.05$). [illegible]wever, no significant differences were observed between dilution rates in pipped chicks and early or late mortality of embryos. The results also explained that DMA concentration negatively affected hatchability, which was higher in 3% and 6% DMA compared to 9% DMA ($p<0.05$). In contrast, DMA concentrations did not influence the other traits ($P > 0.05$). Furthermore, there were significant ($p<0.05$) interaction effects for

dilution rate × DMA concentration on fertility rate, hatchability, and pipped chicks. The LSC × 3% and LSC × 6% groups resulted in 100% hatchability of fertile eggs, albeit with low fertility rates (~6–7%). In contrast, HSC treatments exhibited higher fertility rates (18–26%), but lower hatchability (25–35%) across DMA concentrations. The LSC × 9% group showed 100% pipped chicks but zero hatchability. There were no significant effects of the dilution rates or DMA concentrations on the dead embryos; however, the percentage of early dead embryos tended to increase (p = 0.083) when using a working dilution rate at HSC compared to the LSC group.

Table 2. Effect of semen dilution rate on post-thaw fertility parameters of rooster sperm frozen with various concentrations of dimethyl acetamide.

Treatment Groups	Incubated eggs	Fertile eggs, %[1]	Hatched chicks, %[2]	Pipped chicks, %[2]	Early dead chicks, %[2]	Late dead chicks, %[2]
**Dilution rate**						
**LSC**	255	6.7 ^{b}	64.7 ^{a}	35.3	0.0	0.0
**HSC**	256	22.7 ^{a}	31.0 ^{b}	50.0	15.5	3.4
**p-value**	-	<0.001	0.012	0.285	0.083	0.438
**DMA concentration**						
**3%**	172	16.9	48.3 ^{a}	41.4	6.9	3.4
**6%**	167	12.0	50.0 ^{a}	40.0	10.0	0.0
**9%**	172	15.1	19.2 ^{b}	57.7	19.2	3.8
**p-value**	-	0.437	0.042	0.377	0.354	0.685
**Interaction**						

**LSC × 3%**	85	7.1 [b]	100.0 [a]	0.0 [b]	0.0	0.0
**LSC × 6%**	84	6.0 [b]	100.0 [a]	0.0 [b]	0.0	0.0
**LSC × 9%**	86	7.0 [b]	0.0 [b]	100.0 [a]	0.0	0.0
**HSC × 3%**	87	26.4 [a]	34.8 [b]	52.2 [ab]	8.7	4.3
**HSC × 6%**	83	18.1 [ab]	33.3 [b]	53.3 [ab]	13.3	0.0
**HSC × 9%**	86	23.3 [a]	25.0 [b]	45.0 [ab]	25.0	5.0
**p-value**	-	<0.001	<0.001	0.004	0.328	0.908

Abbreviations: LSC, dilution rate at low sperm concentration; HSC, dilution rate at high sperm concentration; DMA, dimethyl acetamide; [1] Values were calculated as percentages of total incubated eggs. [2] Values were calculated as percentages of fertile eggs. Means with different superscripts, within a parameter and treatment group, are significantly different ($P<0.05$).

Discussion

Most protocols for semen freezing in chickens and other species include a cooling phase before freezing. During this cooling phase, semen dilution is typically used to achieve a final concentration of chemicals and spermatozoa in the mixture of semen and extender. This phase is designed to acclimate the sperm to reduced temperatures, ensuring their survival during freezing [32]. The current study applied two dilution techniques with the semen extender before subjecting it to the cooling process. It compared the effects of HSC versus LSC on sperm motility and fertility traits post-thawing. Sperm concentration and the dilution rate may be related to the post-thaw characteristics of spermatozoa [33].

It is suggested that excessive semen dilution, which logically resulted in a very low sperm concentration, may reduce the overall sperm longevity [34]. The current study showed that the dilution rate at HSC (2×10^9 sperm/mL) significantly increased the rooster sperm TM

and VCL post-thawing, compared to the dilution rate at LSC (1×10^9 sperm/mL). Congruent with these results, Iaffaldano, Angelo (25) found that quality characteristics of post-thawed turkey sperm, such as motility, viability, and osmotic resistance, could be improved linearly by increasing sperm concentration from 0.5 to 4×10^9/mL. However, they found that using a higher sperm concentration of $5\times x10^9$/mL decreased the post-thaw sperm quality. Moreover, previous studies reported that using HSC during a short-term storage technique enhanced sperm motility and viability in the Pearl Guinea fowl [35] and chickens [36]. The observed improvements in general sperm motility of the HSC group can mitigate cryoinjury, likely due to reduced intracellular ice formation and osmotic stress during freezing [37]. Conversely, Zaniboni, Cassinelli (13) found that using LSC at 1×10^9 sperm/mL displayed a higher sperm motility compared to HSC at 1.5×10^9 sperm/mL. In this case, the positive impact of the LSC technique on sperm motility could be interpreted by the effective dilution of cryoprotectant and/or seminal plasma, which hinders the potential of spermatozoa during storage [28, 29, 38].

As shown in Table 1, our results show different kinetic patterns of spermatozoa between the HSC and LSC groups. It was observed that the HSC technique was associated with increased TM and VCL, yet it paradoxically reduced sperm LIN and STR. At the same time, no significant effect of the dilution methodology was observed on the PM and VSL. Increased sperm concentration leads to elevated sperm activation and motility, resulting in a higher proportion of motile sperm and increased path velocity [39]. This is particularly important in species with intense sperm competition, such as many birds, where maximizing motile sperm is advantageous for fertilization success [40]. At higher concentrations, sperm cells are more likely to experience mechanical stimulation through sperm-sperm interactions, mimicking conditions in the female reproductive tract and triggering hyperactivated-like movement patterns characterized by vigorous, nonlinear motion [41]. However, as concentration increases, physical interference between spermatozoa also rises. Frequent collisions hinder

coordinated forward movement, decreasing LIN and STR [39]. Additionally, hydrodynamic forces generated by dense sperm populations can alter the flow environment around cells, disrupting the efficiency of progressive motility and contributing to curvilinear paths [40]. These effects collectively increase the curvilinear velocity (VCL) but reduce the ability of sperm to move in a straight trajectory. This trade-off is particularly notable in avian species such as the domestic fowl (*Gallus gallus*) and ostriches (*Struthio camelus*). For instance, Muvhali, Bonato (41) demonstrated that while total motility and VCL increased with sperm concentration in ostriches, progressive traits like LIN and STR declined. Thus, the observed pattern of high motility but reduced directionality suggests a biological balancing act between maximizing the number of motile sperm and maintaining efficient sperm transport. This factor may critically influence reproductive success in birds and other species with internal fertilization.

The current study demonstrated that fertile eggs in the HSC group were at least 3-fold higher than those obtained from the LSC group. The higher TM, VCL, and ALH observed in HSC samples may contribute to better preservation of flagellar activity, which is critical for fertilizing capacity [37, 42]. It could also be related to spermatozoa's poor antifreeze performance when employing excessively high dilution rates [43]. The concentration of proteins, natural antioxidants, and other advantageous elements in seminal plasma—all necessary for the preservation of the membrane integrity, metabolism, function, and transport of spermatozoa in the female reproductive tract following cryopreservation—is believed to be reduced when semen is excessively diluted [44, 45]. Additionally, too much dilution of semen can damage spermatozoa structure, clump spermatozoa together, drastically reduce the amount of time semen can be preserved, and consequently decrease spermatozoa motility, metabolic activity, and fertilization ability [46], Zhang, Wang (47). Interestingly, despite better motility indices in HSC groups, fertility outcomes showed a divergent pattern. While HSC samples

yielded higher fertilization rates, hatchability of fertile eggs was significantly higher in the LSC groups (Table 2). This discrepancy could be attributed to a possible over-concentration of spermatozoa leading to polyspermy, as suggested in earlier avian reproduction studies [48]. The low hatchability of fertile eggs in the HSC group may also be attributed to the increased embryonic mortality, as indicated in our results (Table 2) and previous studies [49]. Donoghue and Wishart (50) noted that in turkeys and chickens, high sperm concentration during artificial insemination decreased fertilization efficiency due to poor progressive motility, even when total motility remained high. The balance between adequate sperm numbers for fertilization and avoiding excessive spermatozoa appears crucial for optimizing fertilization and subsequent embryo development [50].

Regarding DMA concentration, our results indicated that the intermediate level of 6% consistently provided superior outcomes in both motility and fertility parameters. In consistency with this result, previous studies showed that chicken semen frozen with 6% DMA demonstrated better motility traits when compared to lower or higher DMA concentrations [17, 51, 52]. These results back up earlier studies showing that cryoprotectants, including DMA, exert a concentration-dependent impact on the post-thaw *in vitro* quality of cryopreserved chicken sperm [18, 52] and, if supplemented in moderate concentrations, they balance sperm membrane protection and cytotoxicity [42]. Supplementing DMA at 9% in the current study appeared detrimental, as reflected in reduced motility, hatchability, and increased early embryo mortality, possibly due to toxic effects on sperm membranes and DNA integrity [53]. Conversely, lower DMA (3%) yielded suboptimal motility and fertility, likely due to insufficient cryoprotection.

The significant interaction effects between dilution rate and DMA concentration on various motility and fertility traits underscore the importance of optimizing both factors concurrently. Although HSC combined with 6% DMA achieved the highest motility

parameters, LSC combined with 6% DMA achieved perfect hatchability of fertile eggs, albeit at low fertility rates. At 9% DMA, the LSC group presented a substantial increase in the pipped eggs with a dramatic failure in hatchability, compared to the HSC group, probably due to the high amount of DMA per sperm cell, which exerts a harmful toxic effect on sperm [15, 28, 29]. Indeed, these findings highlight a trade-off, as indicated by [48, 50], that protocols maximizing motility do not necessarily maximize hatchability, and that optimal freezing strategies should consider the intended application (e.g., artificial insemination dose, egg production system).

Conclusions

Our findings suggest that applying high sperm concentration and 6% DMA for chicken semen freezing may best maintain the post-thaw sperm motility and fertility outcomes after artificial insemination. Further studies evaluating sperm functional integrity (e.g., acrosome status, DNA fragmentation) and in vivo fertility trials are warranted to understand the mechanism of action and refine these protocols.

Funding statement

This research was financially supported by the funds from the Academy of Scientific Research and Technology, Egypt (ASRT 5105) and the Vice Presidency for Graduate Studies and Scientific Research at King Faisal University, Saudi Arabia (Project KFU252322).

Acknowledgements

The authors acknowledge the administrative, technical, and financial support of the ASRT staff during this study. They also recognize the Vice Presidency for Graduate Studies

and Scientific Research at King Faisal University for the additional resources and funds provided for this work.

References

1. Blesbois E. Current status in avian semen cryopreservation. World's Poult Sci J 2007;63(2):213-22.
2. Yánez-Ortiz I, Catalán J, Rodríguez-Gil JE, Miró J, Yeste MJARS. Advances in sperm cryopreservation in farm animals: Cattle, horse, pig and sheep. 2022;246:106904.
3. Long J. Avian semen cryopreservation: what are the biological challenges? Poult sci. 2006;85(2):232-6.
4. Sasaki K, Tatsumi T, Tsutsui M, Niinomi T, Imai T, Naito M, et al. A method for cryopreserving semen from Yakido roosters using N-methylacetamide as a cryoprotective agent. The Journal of Poultry Science. 2010:1009030069-.
5. Blesbois E. Freezing avian semen. Avian biology research. 2011;4(2):52-8.
6. Osuga T, Hasegawa T, Aoki M, Fujie M, Nakane T, Asai TJNN. Efficiencies of the cryoprotectants N-Methylacetamide, N-Methylformamide and Dimethyl Sulfoxide, and the cell protectants trehalose and hydroxyethyl starch in cryopreservation of swine sperm. Nanomed Nanotechnol. 2018;3:000139.
7. Wilmut IJARAB. From germ cell preservation to regenerative medicine: an exciting research career in biotechnology. Annu Rev Anim Biosci. 2014;2(1):1-21.
8. Purdy P, Song Y, Silversides F, Blackburn H. Evaluation of glycerol removal techniques, cryoprotectants, and insemination methods for cryopreserving rooster sperm with implications of regeneration of breed or line or both. Poult sci. 2009;88(10):2184-91.
9. Mocé E, Grasseau I, Blesbois E. Cryoprotectant and freezing-process alter the ability of chicken sperm to acrosome react. Anim Reprod Sci. 2010;122(3-4):359-66.
10. Blesbois E, Grasseau I, Seigneurin F. Membrane fluidity and the ability of domestic bird spermatozoa to survive cryopreservation. Reproduction. 2005;129(3):371-8.
11. Partyka A, Niżański W, Łukaszewicz E. Evaluation of fresh and frozen-thawed fowl semen by flow cytometry. Theriogenology. 2010;74(6):1019-27.
12. Blanco JM, Long JA, Gee G, Wildt DE, Donoghue AM. Comparative cryopreservation of avian spermatozoa: Benefits of non-permeating osmoprotectants and ATP on turkey and crane sperm cryosurvival. Anim Reprod Sci. 2011;123(3-4):242-8.
13. Zaniboni L, Cassinelli C, Mangiagalli MG, Gliozzi TM, Cerolini S. Pellet cryopreservation for chicken semen: Effects of sperm working concentration, cryoprotectant concentration, and equilibration time during in vitro processing. Theriogenology. 2014;82(2):251-8.
14. Abouelezz F, Castaño C, Toledano-Díaz A, Esteso M, López-Sebastián A, Campo J, et al. Effect of the interaction between cryoprotectant concentration and cryopreservation method on frozen/thawed chicken sperm variables. Reprod Domest Anim. 2015;50(1):135-41.
15. Abouelezz F, Sayed M, Santiago-Moreno J. Fertility disturbances of dimethylacetamide and glycerol in rooster sperm diluents: Discrimination among effects produced pre and post freezing-thawing process. Anim Reprod Sci. 2017;184:228-34.
16. Mehaisen GM, Partyka A, Ligocka Z, Niżański WJArs. Cryoprotective effect of melatonin supplementation on post-thawed rooster sperm quality. Anim Reprod Sci. 2020;212:106238.

17. Mehaisen GM, Elomda AM, Hamad SK, Ghaly MM, Sun Y, Li Y, et al. Effect of Dimethylacetamide Concentration on Motility, Quality, Antioxidant Biomarkers, Anti-Freeze Gene Expression, and Fertilizing Ability of Frozen/Thawed Rooster Sperm. Animals. 2022;12(20):2739.
18. Hamad SK, Elomda AM, Sun Y, Li Y, Zong Y, Chen J, et al. The In Vitro Evaluation of Rooster Semen Pellets Frozen with Dimethylacetamide. Animals. 2023;13(10):1603. PubMed PMID: doi:10.3390/ani13101603.
19. Elomda AM, Mehaisen GM, Stino FK, Saad MF, Ghaly MM, Partyka A, et al. The characteristics of frozen-thawed rooster sperm using various intracellular cryoprotectants. Poult Sci. 2024:104190.
20. Tang M, Cao J, Yu Z, Liu H, Yang F, Huang S, et al. New semen freezing method for chicken and drake using dimethylacetamide as the cryoprotectant. Poult sci. 2021;100(8):101091.
21. Parker H, McDaniel CJPs. The immediate impact of semen diluent and rate of dilution on the sperm quality index, ATP utilization, gas exchange, and ionic balance of broiler breeder sperm. 2006;85(1):106-16.
22. Parker H, Karaca A, Yeatman J, Frank L, McDaniel CJPs. Fertility of broiler breeders following categorization by the Optibreed® sperm quality index when hens are inseminated with a constant number of sperm. 2002;81(2):239-45.
23. Parker H, McDaniel CJJoapr. Selection of young broiler breeders for semen quality improves hatchability in an industry field trial. 2002;11(3):250-9.
24. Cassinelli C ZL, Mangiagalli MG, Cerolini S, editor Sperm quality changes during equilibration time at 4°C before cryopreservation of chicken semen. Biannual Meeting Association for Applied Animal Andrology; 2008.
25. Iaffaldano N, Angelo M, Mario G, Michele DI, and Pina Rosato M. Effect of different sperm concentrations on the post-thaw viability and motility of turkey spermatozoa cryopreserved by the pellet method. Italian Journal of Animal Science. 2009;8(sup2):760-2. doi: 10.4081/ijas.2009.s2.760.
26. Iaffaldano N, Romagnoli L, Manchisi A, Rosato MP. Cryopreservation of turkey semen by the pellet method: Effects of variables such as the extender, cryoprotectant concentration, cooling time and warming temperature on sperm quality determined through principal components analysis. Theriogenology. 2011;76(5):794-801.
27. Iaffaldano N, Manchisi A, Gambacorta M, Di Iorio M, Pina Rosato M. Effect of different sperm concentrations on the post-thaw viability and motility of turkey spermatozoa cryopreserved by the pellet method. Italian Journal of Animal Science. 2009;8(sup2):760-2.
28. Iaffaldano N, Meluzzi AJT. Effect of dialysis on quality characteristics of turkey semen during liquid storage. 2003;60(3):421-7.
29. Douard V, Hermier D, Labbe C, Magistrini M, Blesbois EJT. Role of seminal plasma in damage to turkey spermatozoa during in vitro storage. 2005;63(1):126-37.
30. Bakst M, Dymond J. Artificial insemination in poultry. Success in Artificial Insemination-Quality of Semen and Diagnostics Employed. 2013:722-3.
31. Siudzińska A, Łukaszewicz EJJoAPR. Effect of semen extenders and storage time on sperm morphology of four chicken breeds. 2008;17(1):101-8.
32. Blank MH, Ruivo LP, Novaes GA, Lemos EC, Losano JD, Siqueira AF, et al. Assessing different liquid-storage temperatures for rooster spermatozoa. 2021;233:106845.
33. Okano T, Murase T, Asano M, Tsubota TJJovms. Effects of final dilution rate, sperm concentration and times for cooling and glycerol equilibration on post-thaw characteristics of canine spermatozoa. 2004;66(11):1359-64.
34. England GJJor, Supplement f. Cryopreservation of dog semen: a review. 1993;47:243-55.
35. Hudson G, Omprakash A, Premavalli KJAJAVA. Research article effect of semen diluents, dilution rates and storage periods on live and abnormal spermatozoa of pearl guinea fowls. 2016;11(7):411-6.
36. Gliozzi T, Zaniboni L, Iaffaldano N, Cerolini S. Spermatozoa DNA and plasma membrane integrity after pellet optimized processing for cryopreservation in meat type chicken breeders. Br Poult Sci. 2017;58(5):578-84.

37. Partyka A, Łukaszewicz E, Niżański W. Effect of cryopreservation on sperm parameters, lipid peroxidation and antioxidant enzymes activity in fowl semen. Theriogenology. 2012;77(8):1497-504.
38. Blesbois E, Brillard J-P. Specific features of in vivo and in vitro sperm storage in birds. Animal. 2007;1(10):1472-81.
39. Tesfay HH, Sun Y, Li Y, Shi L, Fan J, Wang P, et al. Comparative studies of semen quality traits and sperm kinematic parameters in relation to fertility rate between 2 genetic groups of breed lines. 2020;99(11):6139-46.
40. McDiarmid CS, Hurley LL, Le Mesurier M, Blunsden AC, Griffith SCJJoEB. The impact of diet quality on the velocity, morphology and normality of sperm in the zebra finch Taeniopygia guttata. 2022;225(9):jeb243715.
41. Muvhali PT, Bonato M, Malecki IA, Cloete SWJA. Mass sperm motility is correlated to sperm motility as measured by computer-aided sperm analysis (CASA) technology in farmed ostriches. 2022;12(9):1104.
42. Santiago-Moreno J, Bernal B, Pérez-Cerezales S, Castaño C, Toledano-Díaz A, Esteso MC, et al. Seminal plasma amino acid profile in different breeds of chicken: Role of seminal plasma on sperm cryoresistance. 2019;14(1):e0209910.
43. Patil S, Kumar P, Singh G, Bala R, Jerome A, Patil C, et al. 'Semen dilution effect'on sperm variables and conception rate in buffalo. 2020;214:106304.
44. Garner D, Thomas C, Gravance C, Marshall C, DeJarnette J, Allen CJT. Seminal plasma addition attenuates the dilution effect in bovine sperm. 2001;56(1):31-40.
45. Anand M, Yadav S, Kumar A, Vaswani S, Shukla PJJoAR. Effect of dilution and sperm concentration on post thaw semen quality in Barbari buck. 2018;8(4):587-92.
46. Hayden SS, Blanchard TL, Brinsko SP, Varner DD, Hinrichs K, Love CCJT. The "dilution effect" in stallion sperm. 2015;83(4):772-7.
47. Zhang L, Wang X, Jiang C, Sohail T, Sun Y, Sun X, et al. Effect of Different Dilution Methods and Ratios of Ram Semen on Sperm Parameters after Cryopreservation. 2024;14(6):907.
48. Santiago-Moreno J, Blesbois E. Functional aspects of seminal plasma in bird reproduction. International Journal of Molecular Sciences. 2020;21(16):5664.
49. Rakha B, Ansari M, Akhter S, Hussain I, Blesbois E. Cryopreservation of Indian red jungle fowl (Gallus gallus murghi) semen. Anim Reprod Sci. 2016;174:45-55.
50. Donoghue AM, Wishart G. Storage of poultry semen. Anim Reprod Sci. 2000;62(1-3):213-32.
51. Mosca F, Zaniboni L, Sayed AA, Madeddu M, Iaffaldano N, Cerolini SJPS. Effect of dimethylacetamide and N-methylacetamide on the quality and fertility of frozen/thawed chicken semen. Poult Sci. 2019;98(11):6071-7.
52. Zaniboni L, Madeddu M, Mosca F, Sayed AA, Marelli SP, Di Iorio M, et al. Concentration dependent effect of dimethylacetamide and N-methylacetamide on the quality and fertility of cryopreserved chicken semen. Cryobiology. 2022;106:66-72.
53. Rakha B, Ansari M, Akhter S, Akhter A, Blesbois E, Santiago-Moreno J. Effect of dimethylformamide on sperm quality and fertilizing ability of Indian red jungle fowl (Gallus gallus murghi). Theriogenology. 2020;149:55-61.
